# Probing many-body dynamics in a two-dimensional dipolar spin ensemble

**DOI:** 10.1038/s41567-023-01944-5

**Published:** 2023-03-16

**Authors:** E. J. Davis, B. Ye, F. Machado, S. A. Meynell, W. Wu, T. Mittiga, W. Schenken, M. Joos, B. Kobrin, Y. Lyu, Z. Wang, D. Bluvstein, S. Choi, C. Zu, A. C. Bleszynski Jayich, N. Y. Yao

**Affiliations:** 1 Department of Physics, University of California, Berkeley, CA USA; 2 Materials Science Division, Lawrence Berkeley National Laboratory, Berkeley, CA USA; 3 Department of Physics, University of California, Santa Barbara, CA USA; 4 Department of Physics, Harvard University, Cambridge, MA USA; 5 Department of Physics, Washington University, St. Louis, MO USA

**Keywords:** Quantum metrology, Quantum simulation, Magnetic properties and materials, Sensors and biosensors

## Abstract

The most direct approach for characterizing the quantum dynamics of a strongly interacting system is to measure the time evolution of its full many-body state. Despite the conceptual simplicity of this approach, it quickly becomes intractable as the system size grows. An alternate approach is to think of the many-body dynamics as generating noise, which can be measured by the decoherence of a probe qubit. Here we investigate what the decoherence dynamics of such a probe tells us about the many-body system. In particular, we utilize optically addressable probe spins to experimentally characterize both static and dynamical properties of strongly interacting magnetic dipoles. Our experimental platform consists of two types of spin defects in nitrogen delta-doped diamond: nitrogen-vacancy colour centres, which we use as probe spins, and a many-body ensemble of substitutional nitrogen impurities. We demonstrate that the many-body system’s dimensionality, dynamics and disorder are naturally encoded in the probe spins’ decoherence profile. Furthermore, we obtain direct control over the spectral properties of the many-body system, with potential applications in quantum sensing and simulation.

## Abstract (web summary)

Solid-state systems are established candidates to study models of many-body physics but have limited control and readout capabilities. Ensembles of defects in diamond may provide a solution for studying dipolar systems.

## Main

Understanding and controlling the interactions between a single quantum degree of freedom and its environment represents a fundamental challenge within the quantum sciences^1–9^. Typically, one views this challenge through the lens of mitigating decoherence—enabling one to engineer a highly coherent qubit by decoupling it from the environment^2–5,10–12^. However, the environment itself may consist of a strongly interacting many-body system, which naturally leads to an alternate perspective, namely using the decoherence dynamics of the qubit to probe the fundamental properties of the many-body system^6,7,13–18^. Discerning the extent to which such ‘many-body noise’ can provide insight into transport dynamics, low-temperature order and generic correlation functions of an interacting system remains an essential open question.

The complementary goals of probing and eliminating many-body noise have motivated progress in magnetic resonance spectroscopy for decades, including seminal work exploring the decoherence of paramagnetic defects in solids^6,7,13–16,19^. More recently, many of the developed techniques have re-emerged in the study of solid-state spin ensembles containing optically polarizable colour centres. The ability to prepare spin-polarized pure states enables fundamentally new prospects in quantum science, from the exploration of novel phases of matter^20^ to the development of new sensing protocols^21^.

Prospects for optically polarizable spin ensembles in quantum sensing and simulation could be further enhanced by moving to two-dimensional systems, which represents a long-standing engineering challenge for the colour centre community^22–24^. Despite continued advances in fabrication, the stochastic nature of defect generation strongly constrains the systems one can create. The potential rewards are substantial enough to merit repeated engineering efforts: Two-dimensional, long-range interacting spin systems are known to host interesting ground-state phases such as spin liquids^25–27^. Moreover, two-dimensional spin ensembles enable improved sensing capabilities owing to increased coherence times and uniform distance from the target (Supplementary Information).

**Fig. 1:**
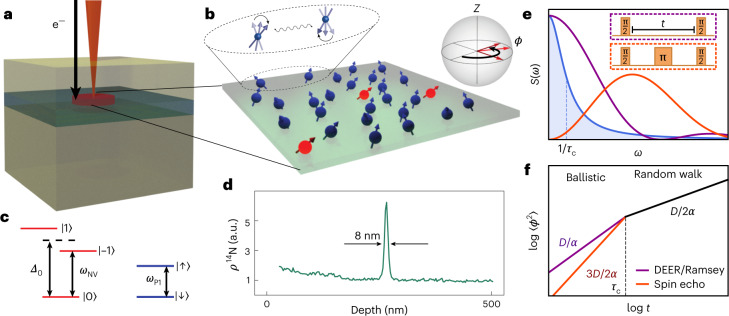
Experimental platform and theoretical framework. **a**, A delta-doped layer of ^14^N (green) is grown on a diamond substrate. NV centres are created via local electron irradiation (orange beam) and subsequent high-temperature annealing. **b**, Schematic depiction of a two-dimensional layer of NV (red) and P1 (blue) centres. Dilute NV centres function as probe spins of the dense, disordered P1 system. The P1 centres exhibit spin-flip dynamics driven by magnetic dipole–dipole interactions (zoom). Ising interactions with the P1 system cause the NV to accumulate a phase, *ϕ*, during noise spectroscopy (Bloch sphere). **c**, NV and P1 level structure in the presence of a magnetic field, *B*, applied along the NV axis. We work within an effective spin 1/2 subspace of the NV centre, $\{\left\vert 0\right\rangle ,\left\vert -1\right\rangle \}$, with level splitting, *ω*_NV_. The corresponding P1 splitting, *ω*_P1_, is strongly off-resonant from the NV transition. **d**, Secondary ion mass spectrometry measurement of the density of ^14^N as a function of depth for sample S1. The presence of a thin layer is indicated by a sharp nitrogen peak with a 8-nm width, limited by the secondary ion mass spectrometry resolution. **e**, The overlap between the many-body spectral function (blue) and the power spectrum of the filter function ∣*f*(*ω*; *t*)∣^2^ determines the variance of the phase ∼ *χ*(*t*) (equation (2)). ∣*f*(*ω*; *t*)∣^2^ for both a Ramsey/DEER pulse sequence (purple) and a spin echo pulse sequence (orange) are shown. **f**, Schematic depiction of the variance of the phase, $\left\langle {\phi }^{2}\right\rangle =-2\log C(t)$, as a function of the measurement duration *t*, for both Ramsey/DEER (purple) and spin echo (orange). The labelled slopes indicate the predicted stretch powers in both the early-time ballistic regime and the late-time random-walk regime (Table 1). The cross-over occurs at the correlation time, *τ*_c_. Source data

In this article, we investigate many-body noise generated by a thin layer of paramagnetic defects in diamond. Specifically, we combine nitrogen delta doping during growth with local electron irradiation to fabricate a diamond sample (S1) where paramagnetic defects are confined to a layer whose width is, in principle, smaller than the average spin defect spacing (Fig. 1a,b)^22–24^. This layer contains a hybrid spin system consisting of two types of defects: spin 1 nitrogen vacancy (NV) centres and spin 1/2 substitutional nitrogen (P1) centres. The dilute NV centres can be optically initialized and read out, making them a natural probe of the many-body noise generated by the strongly interacting P1 centres. In addition, we demonstrate a complementary role for the NV centres, as a source of spin polarization for the optically dark P1 centres. In particular, by using a Hartmann–Hahn protocol, we directly transfer polarization between the two spin ensembles.

We experimentally characterize the P1 system’s many-body noise via the decoherence dynamics of NV probe spins. To elucidate our results, we first present a theoretical framework that unifies and generalizes existing work, predicting a non-trivial temporal profile that exhibits a cross-over between two distinct stretched exponential decays (for the average coherence of the probe spins) (Fig. 1)^6,13–16,19,28^. Beyond solid-state spin systems, the framework naturally extends to a broader class of quantum simulation platforms, including trapped ions, Rydberg atoms and ultracold polar molecules^29^. Crucially, we demonstrate that the associated stretch powers contain a wealth of information about both the static and dynamical properties of the many-body spin system.

We focus on three such properties. First, the stretch power contains a direct signature revealing the dimensionality of the disordered many-body system. Unlike previous work on lower-dimensional ordered systems in magnetic resonance spectroscopy^30–32^, we cannot leverage conventional methods such as X-ray diffraction to characterize our disordered spin ensemble. To the best of our knowledge, studying the decoherence dynamics provides the only robust method to determine the effective dimensionality seen by the spins.

The stretch power of the NV centres’ decoherence can also distinguish between different forms of spectral diffusion, shedding light on the nature of local spin fluctuations. In particular, we demonstrate that the P1 spin-flip dynamics are inconsistent with the conventional expectation of telegraph noise but rather follow that of a Gauss–Markov process (Table 1). Understanding the statistical properties of the many-body noise and the precise physical settings where such noise emerges remains the subject of active debate^4,6,16,33–39^. Finally, the cross-over in time between different stretch powers allows one to extract the many-body system’s correlation time. We demonstrate this behaviour by actively controlling the correlation time of the P1 system via polychromatic driving, building upon techniques previously utilized in broadband decoupling schemes^40^.

**Table 1:** Predicted early- and late-time stretch powers of the probe spin decoherence profile when coupled to a *D*-dimensional system via power-law Ising interactions ∼1/*r*^*α*^. We distinguish between Gaussian and telegraph spin-flip noise in the many-body system, which gives rise to different predictions for the early-time spin echo stretch power

Many-body noise properties	Measurement sequence	Early-time (ballistic regime) stretch power	Late-time (random walk regime) stretch power
	DEER/Ramsey	*D*/*α*	*D*/2*α*
	Spin echo	3*D*/2*α*	*D*/2*α*
	DEER/Ramsey	*D*/*α*	*D*/2*α*
	Spin echo	1 + *D*/*α*	*D*/2*α*

## Theoretical framework for decoherence dynamics induced by many-body noise

We first outline a framework, building upon classic results in NMR spectroscopy, for understanding the decoherence dynamics of probe spins coupled to an interacting many-body system. This will enable us to present a unified theoretical background for understanding the experimental results in subsequent sections^6,14,15,19,28,41–43^. The dynamics of a single probe spin generically depend on three properties: (1) the nature of the system–probe coupling, (2) the system’s many-body Hamiltonian *H*_int_ and (3) the measurement sequence itself. Crucially, by averaging across the dynamics of many such probe spins, one can extract global features of the many-body system (Fig. 1b). We distinguish between two types of ensemble averaging that give rise to distinct signatures in the decoherence: (1) an average over many-body trajectories (that is, both spin configurations and dynamics) that yields information about the microscopic spin fluctuations (for simplicity, we focus our discussion on the infinite-temperature limit, but the analysis can be extended to finite temperature) and (2) an average over positional randomness (that is, random locations of the system spins) that yields information about both dimensionality and disorder.

To be specific, let us consider a single spin 1/2 probe coupled to a many-body ensemble via long-range, 1/*r*^*α*^ Ising interactions:

1 $${H}_{z}=\mathop{\sum}\limits_{i}\frac{{J}_{z}}{{r}_{i}^{\alpha }}{\hat{s}}_{\mathrm{p}}^{z}{\hat{s}}_{i}^{z},$$

where *r*_*i*_ is the distance between the probe spin ${\hat{s}}_{\mathrm{p}}$ and the *i*th system spin ${\hat{s}}_{i}$, and the Ising coupling strength *J*_*z*_ implicitly includes any angular dependence. Such power-law interactions are ubiquitous in solid-state, atomic and molecular quantum platforms (for example, Ruderman–Kittel–Kasuya–Yosida interactions, electric/magnetic dipolar interactions, van der Waals interactions, etc.).

Physically, the system spins generate an effective magnetic field at the location of the probe (via Ising interactions), which can be measured with Ramsey spectroscopy (Fig. 1e, inset)^7^. In particular, we envision initially preparing the probe in an eigenstate of ${\hat{s}}_{\mathrm{p}}^{z}$ and subsequently rotating it with a π/2 pulse such that the initial normalized coherence is unity, $C\equiv 2\left\langle {\hat{s}}_{\mathrm{p}}^{x}\right\rangle =1$. The magnetic field, which fluctuates due to many-body interactions, causes the probe to Larmor precess (Fig. 1b, inset and Supplementary Information). The phase associated with this Larmor precession can be read out via a population imbalance, after a second π/2 pulse.

### Average over many-body trajectories

For a many-body system at infinite temperature, $C(t)=2{{{\rm{Tr}}}}[\rho (t){\hat{s}}_{\mathrm{p}}^{x}]$, where *ρ*(*t*) is the full density matrix that includes both the system and the probe. The spin fluctuations are determined by the microscopic details of the many-body dynamics whose full analysis is intractable. To make progress, we approximate each spin as a stochastic classical variable ${\hat{s}}_{i}^{z}(t)\to {s}_{i}^{z}(t)$. The statistical properties of such variables and their resulting ability to capture the experimental observations provide important insights into the nature of fluctuations in strongly interacting spin systems.

The phase of the Larmor precession is given by $$\phi (t)= \int\nolimits_{0}^{t}\mathrm{d}$$$${t}^{{\prime} }\,{J}_{z}{\sum }_{i}{s}_{i}^{z}({t}^{{\prime} })/{r}_{i}^{\alpha }$$. Assuming that *ϕ*(*t*) is Gaussian distributed, one finds that the average probe coherence decays exponentially as $$C(t)=\left\langle {{{\rm{Re}}}}[\mathrm{e}^{-\mathrm{i}\phi (t)}]\right\rangle \approx \mathrm{{e}}^{-\left\langle {\phi }^{2}\right\rangle /2}$$, where $$\left\langle {\phi }^{2}\right\rangle = {\sum }_{i}{J}_{z}^{2}\chi (t)/{r}_{i}^{2\alpha }$$ (refs. ^4,13,39,44^; see the Supplementary Information for supporting derivations). Here, *χ*(*t*) encodes the response of the probe spins to the noise spectral density, *S*(*ω*), of the many-body system:

2 $$\chi (t)\equiv \int\,\mathrm{d}\omega \,| f(\omega ;t){| }^{2}S(\omega ),$$

where *f*(*ω*; *t*) is the filter function associated with a particular pulse sequence (for example, Ramsey spectroscopy or spin echo) of total duration *t* (Fig. 1e).

Intuitively, *S*(*ω*) quantifies the noise power density of spin flips in the many-body system. It is the Fourier transform of the autocorrelation function, $\xi (t)\equiv 4\left\langle {s}_{i}^{z}(t){s}_{i}^{z}(0)\right\rangle$, and captures the spin dynamics at the level of two-point correlations^45^. For Markovian dynamics, $\xi (t)={\mathrm{e}}^{-| t| /{\tau }_{\mathrm{c}}}$, where *τ*_*c*_ defines the correlation time after which a spin, on average, retains no memory of its initial orientation. In this case, *S*(*ω*) is Lorentzian and one can derive an analytic expression for *χ* (refs. ^15,19,37,41^; see the Supplementary Information for supporting derivations).

A few remarks are in order. First, the premise that many-body Hamiltonian dynamics produce Gaussian-distributed phases *ϕ*(*t*)—while often assumed—is challenging to analytically justify^6,15,16,33,46^. Indeed, a well-known counterexample of non-Gaussian spectral diffusion occurs when the spin dynamics can be modelled as telegraph noise, that is, stochastic jumps between discrete values ${s}_{i}^{z}=\pm {s}_{i}$ (refs. ^16,34^). The precise physical settings where such noise emerges remain the subject of active debate^4,6,16,33–39^. Second, we note that our Markovian assumption is not necessarily valid for a many-body system at early times or for certain forms of interactions, which can also affect the decoherence dynamics.

### Average over positional randomness

The probe’s decoherence depends crucially on the spatial distribution of the spins in the many-body system. For disordered spin ensembles, explicitly averaging over their random positions yields a decoherence profile:

3 $$C(t)=\int\mathop{\prod }\limits_{i=1}^{N}\frac{{\mathrm{d}}^{D}{r}_{i}}{V}\exp \left[\frac{-{J}_{z}^{2}\chi (t)}{2{r}_{i}^{2\alpha }}\right]={\mathrm{e}}^{-an{[{J}_{z}^{2}\chi (t)]}^{D/2\alpha }},$$

where *a* is a dimensionless constant and *N* is the number of system spins in a *D*-dimensional volume *V* at a density *n* ≡ *N*/*V* (see the Supplementary Information for supporting derivations)^19^. By contrast, for spins on a lattice or for a single probe spin, the exponent of the coherence scales as $\sim {J}_{z}^{2}\chi (t)$ (Supplementary Information).

A resonance counting argument underlies the appearance of both the dimensionality and the interaction power law in equation (3). Roughly, a probe spin is only coupled to system spins that induce a phase variance larger than some cutoff *ϵ*. This constraint on the minimum variance defines a volume of radius ${r}_{\max } \approx {[\,{J}_{z}^{2}\chi (t)/\epsilon ]}^{1/2\alpha }$ containing ${N}_{\mathrm{s}} \approx n{r}_{\max }^{D}$ spins, implying that the total variance accrued at any given time is $\epsilon {N}_{\mathrm{s}} \approx {[\,{J}_{z}^{2}\chi (t)]}^{D/2\alpha }$. Thus, the positional average simply serves to count the number of spins to which the probe is coupled.

### Decoherence profile

The functional form of the probe’s decoherence, *C*(*t*), encodes a number of features of the many-body system. We begin by elucidating them in the context of Ramsey spectroscopy. First, one expects a somewhat sharp cross-over in the behaviour of *C*(*t*) at the correlation time *τ*_c_. For early times, *t* ≪ *τ*_c_, the phase variance accumulates as in a ballistic trajectory with *χ* ∼ *t*^2^, while for late times, *t* ≫ *τ*_c_, the variance accumulates as in a random walk with *χ* ∼  *t* (refs. ^15,28,41^). This leads to a simple prediction, namely that the stretch power, *β*, of the probe’s exponential decay (that is, $-\log C \propto \langle \phi^2 \rangle \sim t^\beta$) changes from *D*/*α* to *D*/2*α* at the correlation time (Fig. 1f).

Second, moving beyond Ramsey measurements by changing the filter function, one can probe more subtle properties of the many-body noise. In particular, a spin echo sequence filters out the leading-order DC contribution from the many-body noise spectrum, allowing one to investigate higher-frequency correlations of the spin-flip dynamics. Different types of spin-flip dynamics naturally lead to different phase distributions. For the case of Gaussian noise, one finds that (at early times) *χ*  ∼  *t*^3^. However, in the case of telegraph noise, the analysis is more subtle since higher-order moments of *ϕ*(*t*) must be taken into account. This leads to markedly different early-time predictions for *β*, dependent on both the measurement sequence as well as the many-body noise (Table 1).

At late times, however, one expects the probe’s coherence to agree across different pulse sequences and spin-flip dynamics. For example, in the case of spin echo, the decoupling π pulse (Fig. 1e, inset) is ineffective on timescales larger than the correlation time, since the spin configurations during the two halves of the free evolution are completely uncorrelated. Moreover, this same loss of correlation implies that the phase accumulation is characterized by incoherent Gaussian diffusion, regardless of the specific nature of the spin dynamics (for example, Markovian versus non-Markovian or continuous versus telegraph).

## Experimentally probing many-body noise in strongly interacting spin ensembles

Our experimental samples contain a high density of spin 1/2 P1 centres (Fig. 1b, blue spins) which form a strongly interacting many-body system coupled via magnetic dipole–dipole interactions:

4 $${H}_{{{{\rm{int}}}}}=\mathop{\sum}\limits_{i < j}\frac{{J}_{0}}{{r}_{ij}^{3}}\left[{c}_{ij}({\hat{s}}_{i}^{+}{\hat{s}}_{j}^{-}+{\hat{s}}_{i}^{-}{\hat{s}}_{j}^{+})+{\tilde{c}}_{ij}{\hat{s}}_{i}^{z}{\hat{s}}_{j}^{z}\right],$$

where *J*_0_ = 2π × 52 MHz nm^3^, *r*_*ij*_ is the distance between P1 spins *i* and *j* and $c,\tilde{c}$ capture the angular dependence of the dipolar interaction (Supplementary Information). We note that *H*_int_ contains only the energy-conserving terms of the dipolar interaction.

The probes in our system are spin 1 NV centres, which can be optically initialized to $\left\vert {m}_{\mathrm{s}}=0\right\rangle$ using 532 nm laser light. An applied magnetic field *B* along the NV axis splits the $\left\vert {m}_{\mathrm{s}}=\pm 1\right\rangle$ states, allowing us to work within the effective spin 1/2 manifold $\{\left\vert 0\right\rangle ,\left\vert -1\right\rangle \}$. Microwave pulses at frequency *ω*_NV_ are used to perform coherent spin rotations (that is, for Ramsey spectroscopy or spin echo) within this manifold (Fig. 1c).

Physically, the NV and P1 centres are also coupled via dipolar interactions. However, for a generic magnetic field strength, they are highly detuned, that is, ∣*ω*_NV_ − *ω*_P1_∣ is on the order of gigahertz, owing to the zero-field splitting of the NV centre (Δ_0_ = 2π × 2.87 GHz) (Fig. 1c). Since typical interaction strengths in our system are on the order of megahertz, direct polarization exchange between an NV and P1 is strongly off-resonant. The strong suppression of spin-exchange interactions between NV and P1 centres simplifies the full magnetic dipole–dipole Hamiltonian to a system–probe Ising coupling of precisely the form given by equation (1) with *α* = 3 (Supplementary Information).

### Delta-doped sample fabrication

Sample S1 was grown via homoepitaxial plasma-enhanced chemical vapour deposition using isotopically purified methane (99.999% ^12^C)^22^. The delta-doped layer was formed by introducing natural-abundance nitrogen gas during growth (5 sccm, 10 min) in between nitrogen-free buffer and capping layers. To create the vacancies necessary for generating NV centres, the sample was electron irradiated with a transmission electron microscope set to 145 keV (ref. ^23^) and subsequently annealed at 850 °C for 6 h.

### Two-dimensional spin dynamics

**Fig. 2:**
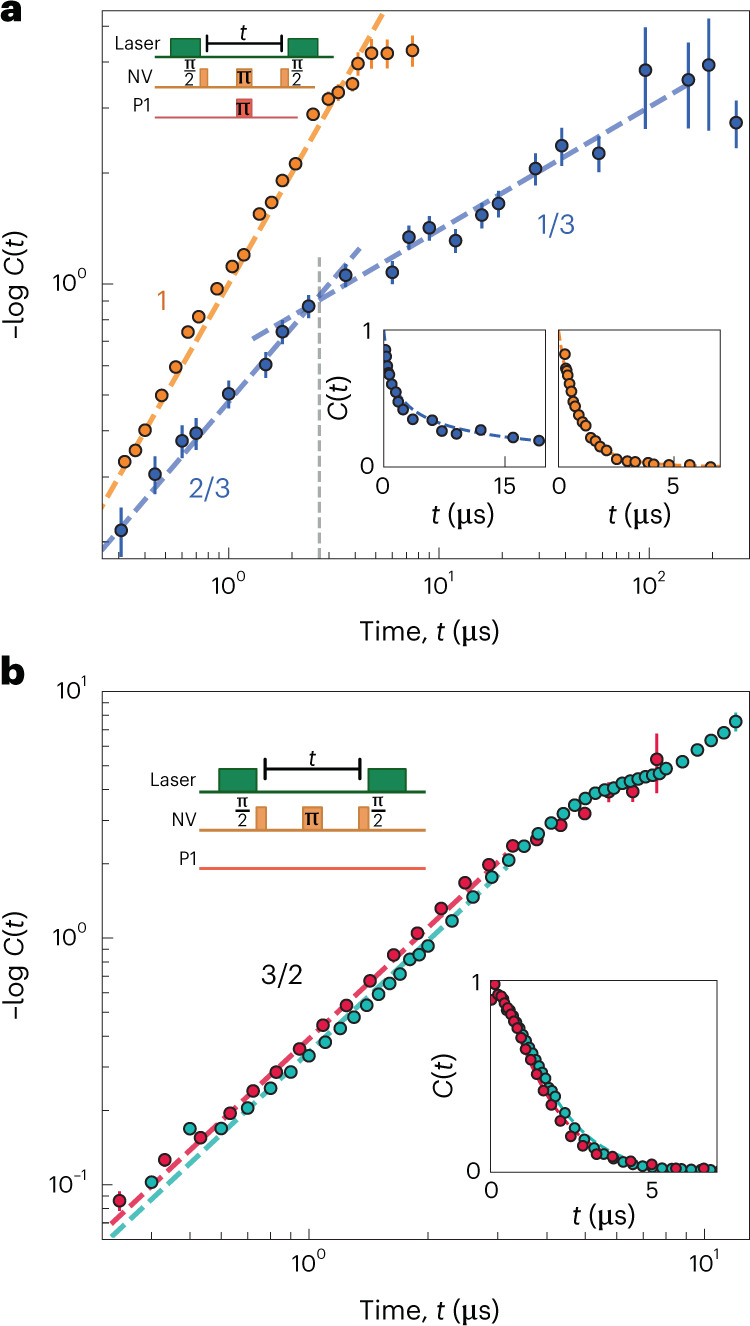
Dimensionality and dynamics from many-body noise. **a**, The normalized coherence for a DEER measurement on sample S1 (blue) and sample S2 (yellow) as a function of the free evolution time *t*. Dashed blue lines indicate the predicted early- and late-time stretch powers of 2/3 and 1/3, respectively, for a dipolar spin system in two dimensions. The dashed yellow line depicts the predicted early-time stretch power of 1 for a dipolar spin system in three dimensions (Table 1). Together, these data demonstrate the two- and three-dimensional nature of samples S1 and S2, respectively. The lower right insets show the same data on a linear scale. The top left inset shows the DEER pulse sequence. **b**, Spin echo measurements on three-dimensional dipolar spin ensembles in samples S3 (teal) and S4 (red) clearly exhibit a stretch power of 3/2 (dotted lines) over nearly two decades in time. This is consistent with the presence of Gaussian noise and allows one to explicitly rule out telegraph noise. The lower right inset shows the same data on a linear scale. The top left inset shows the spin echo pulse sequence. All data are presented as mean ± s.e.m. Source data

We begin by performing double electron-electron resonance (DEER) measurements on sample S1. While largely analogous to Ramsey spectroscopy (Table 1), DEER has the technical advantage that it filters out undesired quasi-static fields (for example, from hyperfine interactions between the NV and host nitrogen nucleus)^7,24^. As shown in Fig. 2a (inset, blue data), the NV’s coherence decays on a timescale of approximately 5 μs.

To explore the functional form of the probe NV’s decoherence, we plot the negative logarithm of the coherence, $-\log C(t)$, on a log–log scale, such that the stretch power, *β*, is simply given by the slope of the data. At early times, the data exhibit *β* = 2/3 for over a decade in time (Fig. 2a, blue data). At a timescale of approximately 3 μs (vertical dashed line), the data cross over to a stretch power of *β* = 1/3 for another decade in time. This behaviour is in excellent agreement with that expected for two-dimensional spin dynamics driven by dipolar interactions (Fig. 1f and Table 1).

For comparison, we perform DEER spectroscopy on a conventional three-dimensional NV–P1 system (sample S2; Methods). As shown in Fig. 2a (orange), the data exhibit *β* = 1 for a decade in time, consistent with the prediction for three-dimensional dipolar interactions (Table 1). However, the cross-over to the late-time ‘random walk’ regime is difficult to experimentally access because the larger early-time stretch power causes a faster decay to the noise floor.

### Characterizing microscopic spin-flip dynamics

To probe the nature of the microscopic spin-flip dynamics in our system, we perform spin echo measurements on three dimensional samples (S3 and S4 (type IB)), which exhibit a much higher P1-to-NV density ratio (Methods). For lower relative densities (that is, samples S1 and S2), the spin echo measurement contains a confounding signal from interactions between the NVs themselves (Methods).

In both samples (S3 and S4), we find that the coherence exhibits a stretched exponential decay with *β* = 3/2 for well over a decade in time (Fig. 2b). Curiously, this is consistent with Gaussian spectral diffusion where *β* = 3*D*/2*α* = 3/2 but patently inconsistent with the telegraph noise prediction of *β* = 1 + *D*/*α* = 2. While in agreement with prior measurements on similar samples^38^, this observation is actually rather puzzling and related to a question in the context of dipolar spin noise^4,6,7,13–16,19,28,33–39,47–49^. In particular, one naively expects that spins in a strongly interacting system should be treated as stochastic binary variables, thereby generating telegraph noise. For the specific case of dipolar spin ensembles, this expectation dates back to seminal work from Klauder and Anderson^6^. The intuition behind this noise model is most easily seen in the language of the master equation—each individual spin ‘sees’ the remaining system as a Markovian bath. The resulting local spin dynamics are then characterized by a series of stochastic quantum jumps that flip the spin orientation and give rise to telegraph noise. Alternatively, in the Heisenberg picture, the same intuition can be understood from the spreading of the operator ${\hat{s}}_{i}^{z}$. This spreading hides local coherences in many-body correlations, leading to an ensemble of telegraph-like, classical trajectories (Supplementary Information).

We conjecture that the observation of Gaussian spectral diffusion in our system is related to the presence of disorder, which strongly suppresses operator spreading^50^. To illustrate this point, consider the limiting case where the operator dynamics are constrained to a single spin. In this situation, the dynamics of ${\hat{s}}_{i}^{z}(t)$ follow a particular coherent trajectory around the Bloch sphere and the rate at which the probe accumulates phase is continuous. Averaging over different trajectories of the coherent dynamics naturally leads to Gaussian noise.

### Controlling the P1 spectral function

**Fig. 3:**
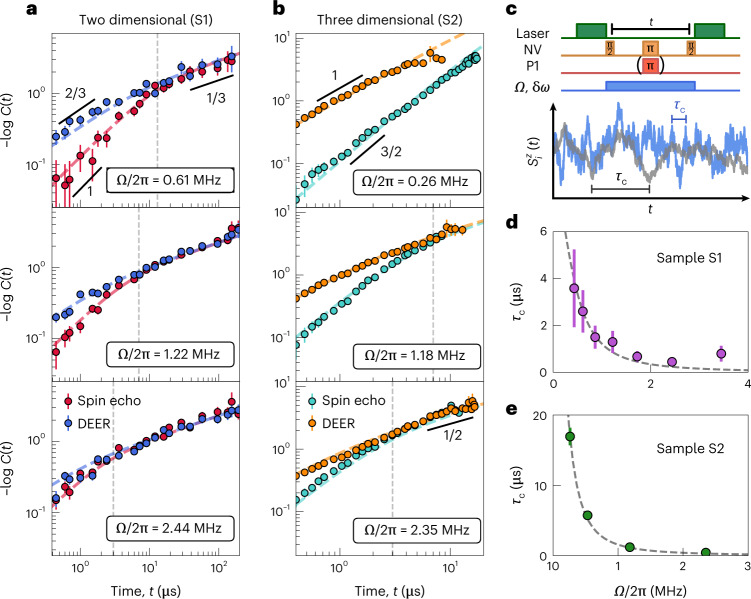
Tuning the correlation time of the bath. **a**,**b**, Measurements of DEER (blue and orange) and spin echo (red and teal) on two- and three-dimensional samples (S1 and S2) for different powers of the polychromatic (that is, incoherent) drive at fixed linewidth δ*ω* of 2π × 18 MHz and 2π × 20 MHz, respectively. The time at which the two signals overlap (vertical dashed lines) acts as a proxy for the correlation time and decreases as the power of the incoherent driving increases (top to bottom panels). The data are well fitted by analytic expressions for [*χ*(*t*)]^*D*/2*α*^ (equation (5)) (dashed curves). Data are presented as mean ± s.e.m. **c**, An incoherent drive field (light blue) with power ∼*Ω*^2^ and linewidth δ*ω* is applied to the P1 spins during the free evolution time *t* of both DEER and spin echo sequences to tune the correlation time of the many-body system. In this case, ${s}_{i}^{z}(t)$ evolves as a Gaussian random process [schematic for short (blue) and long (grey) correlation time *τ*_c_]. **d**,**e**, The correlation times, *τ*_c_, extracted from fitting the data to equation (5) for samples S1 (purple) and S2 (green) plotted as a function of *Ω*, agreeing well with a simple theoretical model (dashed grey curves). Data presented as best fit values ± fitting error. Source data

Next, we demonstrate the ability to directly control the P1 noise spectrum for both two- and three-dimensional dipolar spin ensembles (that is, samples S1 and S2). In particular, we engineer the shape and linewidth of *S*(*ω*) by driving the P1 system with a polychromatic microwave tone^40^. This drive is generated by adding phase noise to the resonant microwave signal at *ω*_P1_ to produce a Lorentzian drive spectrum with linewidth δ*ω* (Fig. 3c). While such techniques originated in the context of broadband noise decoupling^40^, here we directly tune the correlation time of the P1 system and measure a corresponding change in the cross-over timescale between coherent and incoherent spin dynamics^15,49^.

Microscopically, the polychromatic drive leads to a number of physical effects. First, tuning the Rabi frequency, *Ω*, of the drive provides a direct knob for controlling the correlation time, *τ*_c_, of the P1 system. Second, since the many-body system inherits the noise spectrum of the drive, one has provably Gaussian statistics for the spin variables ${s}_{i}^{z}$ (Supplementary Information). Third, our earlier Markovian assumption is explicitly enforced by the presence of a Lorentzian noise spectrum. Taking these last two points together allows one to analytically predict the precise form of the NV probe’s decoherence profile, $-\log C(t) \sim \chi {(t)}^{D/2\alpha }$, for either DEER or spin echo spectroscopy:

5 $$\begin{array}{l}{\chi }^{{{{\rm{DEER}}}}}(t)=2{\tau }_{\mathrm{c}}t-2{\tau }_{\mathrm{c}}^{2}\left(1-{{{{\rm{e}}}}}^{-\frac{t}{{\tau }_{\mathrm{c}}}}\right),\\ {\chi }^{{{{\rm{SE}}}}}(t)=2{\tau }_{\mathrm{c}}t-2{\tau }_{\mathrm{c}}^{2}\left(3+{{{{\rm{e}}}}}^{-\frac{t}{{\tau }_{\mathrm{c}}}}-4{{{{\rm{e}}}}}^{-\frac{t}{2{\tau }_{\mathrm{c}}}}\right).\end{array}$$

We perform both DEER and spin echo measurements as a function of the power (∼*Ω*^2^) of the polychromatic drive for our two-dimensional sample (S1) (Fig. 3a). As expected, for weak driving (Fig. 3a, top), the DEER signal (blue) is analogous to the undriven case, exhibiting a cross-over from a stretch power of *β* = 2/3 at early times to a stretch power of *β* = 1/3 at late times. For the same drive strength, the spin echo data (red) also exhibit a cross-over between two distinct stretch powers, with the key difference being that *β* = 3*D*/2*α* = 1 at early times. This represents an independent (spin echo based) confirmation of the two-dimensional nature of our delta-doped sample.

Recall that, at late times (that is *t* ≳ *τ*_*c*_), one expects the NV’s coherence *C*(*t*) to agree across different pulses sequences (Fig. 1f). This is indeed borne out by the data (Fig. 3). In fact, the location of this late-time overlap provides a proxy for estimating the correlation time and is shown as the dashed grey lines in Fig. 3a. As one increases the power of the drive (Fig. 3a), the noise spectrum, *S*(*ω*), naturally broadens. In the data, this manifests as a shortened correlation time, with the location of the DEER/echo overlap shifting to earlier timescales (Fig. 3a).

Analogous measurements on a three-dimensional spin ensemble (sample S2) reveal much the same physics (Fig. 3b), with stretch powers again consistent with a Gauss–Markov prediction (Table 1). For weak driving, *C*(*t*) is consistent with the early-time ballistic regime for over a decade in time (Fig. 3b, top). However, it is difficult to access late enough timescales to observe an overlap between DEER and spin echo. Crucially, by using the drive to push to shorter correlation times, we can directly observe the late-time random-walk regime in three dimensions, where *β* = 1/2 (Fig. 3b, middle and bottom).

Remarkably, as evidenced by the dashed curves in Fig. 3a,b our data exhibit excellent agreement—across different dimensionalities, drive strengths and pulse sequences—with the analytic predictions presented in equation (5). Moreover, by fitting *χ*^*D*/2*α*^ simultaneously across spin echo and DEER datasets for each *Ω*, we quantitatively extract the correlation time, *τ*_c_. Up to an ${{{\mathcal{O}}}}(1)$ scaling factor, we find that the extracted *τ*_c_ agrees well with the DEER/echo overlap time. In addition, the behaviour of *τ*_c_ as a function of *Ω* also exhibits quantitative agreement with an analytic model that predicts *τ*_c_  ∼  δ*ω*/*Ω*^2^ in the limit of strong driving (Fig. 3d,e).

We emphasize that, although one observes *β* = 3*D*/2*α* in both the driven (Fig. 3a,b) and undriven (Fig. 2b) spin echo measurements, the underlying physics is extremely different. In the latter case, Gaussian spectral diffusion emerges from isolated, disordered, many-body dynamics, while in the former case, it is imposed by the external drive.

## A two-dimensional solid-state platform for quantum simulation and sensing

Our platform offers two distinct paths towards quantum simulation and sensing using strongly interacting, two-dimensional, spin-polarized ensembles. First, treating the NV centres themselves as the many-body system directly leverages their optical polarizability. However, given their relative diluteness, it is natural to ask whether one can access regimes where the NV–NV interactions dominate over other energy scales. Conversely, treating the P1 centres as the many-body system takes advantage of their higher densities and interaction strengths, with the key challenge being that these dark spins cannot be optically pumped. Here, we demonstrate that both of these paths are viable for sample S1: (1) we show that the dipolar interactions among NV centres can dominate their decoherence dynamics, using advanced dynamical decoupling sequences, and (2) we demonstrate direct polarization exchange between NV and P1 centres, providing a mechanism to spin polarize the P1 system.

### Interacting NV ensemble

**Fig. 4:**
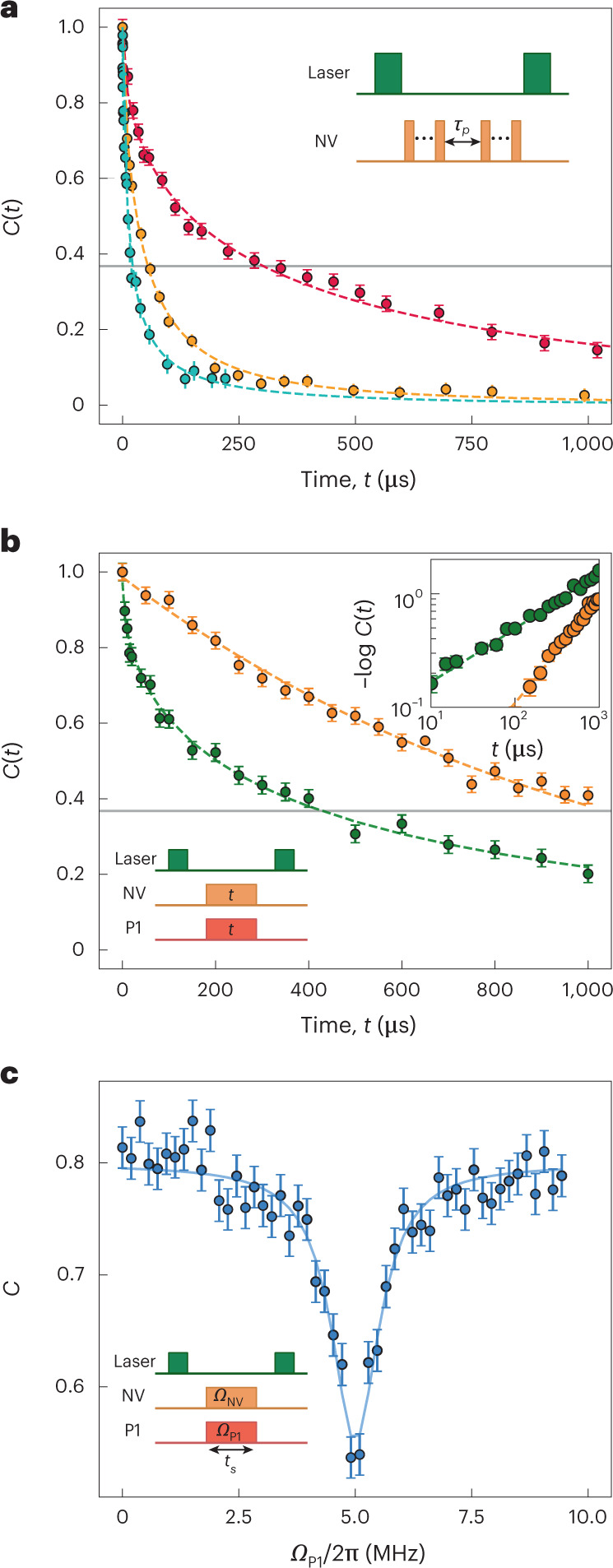
Hybrid two-dimensional spin system for simulation and sensing. **a**, We measure *T*_2_ with spin echo (blue), XY-8 (orange) and DROID (red) pulse sequences with interpulse spacing of *τ*_p_ = 100 ns. The spin echo and XY-8 decoherence profiles are fitted by equation (5), while DROID is fitted by a stretched exponential $\sim {\mathrm{e}}^{-{(t/{T}_{2})}^{1/2}}.$ The 1/e lifetimes (grey line) for spin echo, XY-8 and DROID are 22 μs, 47 μs and 302 μs, respectively. Inset: schematic of dynamical decoupling sequence with interpulse spacing *τ*_p_. **b**, Spin-locked NV depolarization profile. Only NV centres (orange) or both NV and P1 centres (green) are driven at a Rabi frequency of 2π × 5 MHz. The resonant spin-exchange interactions reduce the spin-locking relaxation time *T*_1*ρ*_ by a factor of 3. The top inset shows the same data plotted on a log–log scale to elucidate stretch powers. Bottom inset: spin-locking pulse sequence. **c**, Hartmann–Hahn polarization exchange resonance. The NV Rabi frequency *Ω*_NV_ is fixed at 2π × 5 MHz. When the P1 Rabi frequency *Ω*_P1_ matches *Ω*_NV_, a reduction in contrast is induced by the resonant polarization exchange between NV and P1 centres. The data are fitted by a Lorentzian (dashed curve) with a linewidth of 2π × 1.2 MHz. Inset: Hartmann–Hahn pulse sequence, with fixed spin-locking duration *t*_s_ = 200 μs. All data presented as mean ± s.e.m. Source data

To demonstrate NV–NV interaction-dominated dynamics, we compare the decoherence timescales between spin echo, XY-8 and disorder-robust interaction decoupling (DROID) dynamical decoupling sequences^51^. The spin echo effectively decouples static disorder, while the XY-8 sequence further decouples NV–P1 interactions. As depicted in Fig. 4a, XY-8 pulses extend the spin echo decay time (defined as the 1/e time) by approximately a factor of two. With NV–P1 interactions decoupled, our hypothesis is that the dynamics are now driven by dipolar interactions between the NV centres. To test this, we perform a DROID decoupling sequence, which eliminates the dipolar dynamics between NV centres^51^ (Methods). Remarkably, this extends the coherence time by nearly an order of magnitude, demonstrating that NV–NV interactions are, by far, the dominant source of many-body dynamics in this regime. Moreover, the XY-8 decoherence thus provides an estimate of an average NV spin–spin spacing of 15 nm.

### Interacting P1 ensemble

The polarization of the optically dark P1 ensemble can be realized by either (1) working at low temperatures and large magnetic fields^52^ or (2) using NV centres to transfer polarization to the P1 centres. Here, we focus on the latter. While NV–P1 polarization transfer has previously been demonstrated^53,54^, it has not been measured in a two-dimensional system. Indeed, conjectures about localization in such systems indicate that polarization transfer could be highly suppressed^55,56^.

To investigate, we employ a Hartmann–Hahn sequence designed to transfer polarization between NV and P1 spins in the rotating frame^53,54^. In particular, we drive the NV and P1 spins independently, with Rabi frequencies *Ω*_NV_ and *Ω*_P1_. When only the NV centres are driven, we are effectively performing a so-called spin-locking measurement^57^. For *Ω*_NV_ = 2π × 5 MHz, we find that the NV centres depolarize on a timescale of *T*_1*ρ*_ = 1.05(3) ms (Fig. 4b, orange). The data are cleanly fit by a simple exponential and consistent with phonon-limited decay (Fig. 4b, inset). By contrast, when the driving satisfies the Hartmann–Hahn condition, *Ω*_NV_ = *Ω*_P1_, the NV and P1 spins can resonantly exchange polarization. To characterize this, we fix *Ω*_NV_ = 2π × 5 MHz and choose a spin-locking duration *t*_s_ = 200 μs. By sweeping the P1 Rabi frequency, we indeed observe a resonant polarization exchange feature centred at *Ω*_P1_ = 2π × 5 MHz with a linewidth of ∼ 2π × 1.2 MHz (Fig. 4c), consistent with the intrinsic P1 linewidth. As illustrated in Fig. 4b, on resonance, the NV depolarization is notably enhanced via polarization transfer to the P1 centres and the data exhibit a three-fold decrease in the decay time. Moreover, the data are well fitted with a stretch power of *β* = 1/3 (Fig. 4b, inset), which is also indicative of interaction-dominated decay^47^ (Methods).

## Conclusion and outlook

Our results demonstrate the diversity of information that can be accessed via the decoherence dynamics of a probe spin ensemble. For example, we shed light on a long-standing debate about the nature of spin-flip noise in a strongly interacting dipolar system^4,6,16,33–39,48,49^. Moreover, we directly measure the correlation time of the many-body system and introduce a technique to probe its dimensionality. This technique is particularly useful for disordered spin ensembles embedded in solids^58,59^, where a direct, non-destructive measurement of nanoscale spatial properties is challenging with conventional toolsets.

One can imagine generalizing our work in a number of promising directions. First, the ability to fabricate and characterize strongly interacting, two-dimensional dipolar spin ensembles opens the door to a number of intriguing questions within the landscape of quantum simulation. Indeed, dipolar interactions in 2D are quite special from the perspective of localization, allowing one to experimentally probe the role of many-body resonances^55,56^. In the context of ground-state physics, the long-range, anisotropic nature of the dipolar interaction has also been predicted to stabilize a number of exotic phases, ranging from supersolids to spin liquids^25,26^. Connecting this latter point back to noise spectroscopy, one could imagine tailoring the probe’s filter function to distinguish between different types of ground-state order.

Second, dense ensembles of two-dimensional spins also promise a number of unique advantages with respect to quantum sensing^21,22,24^. For example, a 2D layer of NVs fabricated near the diamond surface would exhibit a pronounced enhancement in spatial resolution (set by the depth of the layer) compared with a three-dimensional ensemble at the same density, *ρ* (refs. ^22,60^). In addition, for samples where the coherence time is limited by spin–spin interactions, a lower dimensionality reduces the coordination number and leads to an enhanced *T*_2_ scaling as *n*^−*α*/*D*^ (Supplementary Information).

Third, one can probe the relationship between operator spreading and Gauss–Markov noise by exploring samples with different relaxation rates, interaction power laws, disorder strengths and spin densities^33,49^. One could also utilize alternate pulse sequences, such as stimulated echo, to provide a more fine-grained characterization of the many-body noise (for example, the entire spectral diffusion kernel)^28,33^.

Finally, our framework can also be applied to long-range-interacting systems of Rydberg atoms, trapped ions and polar molecules. In such systems, the ability to perform imaging and quantum control at the single-particle level allows for greater freedom in designing methods to probe many-body noise. As a particularly intriguing example, one could imagine a non-destructive, time-resolved generalization of many-body noise spectroscopy, where one repeatedly interrogates the probe without projecting the many-body system.

## Methods

### Sample preparation and characterization

#### Sample S1

*Sample fabrication*

Here, we add to the details provided in Section Delta-doped sample fabrication. Sample S1 was grown on a commercially available Element-6 electronic grade (100) substrate, polished by Syntek^61^ to a surface roughness less than 200 pm. Throughout the plasma-enhanced chemical vapour deposition growth process^22^, we used 400 sccm of hydrogen gas with a background pressure of 25 Torr, and a microwave power of 750 W. The sample temperature was held at 800 °C.

*NV density*

**Extended Data Fig. 1:**
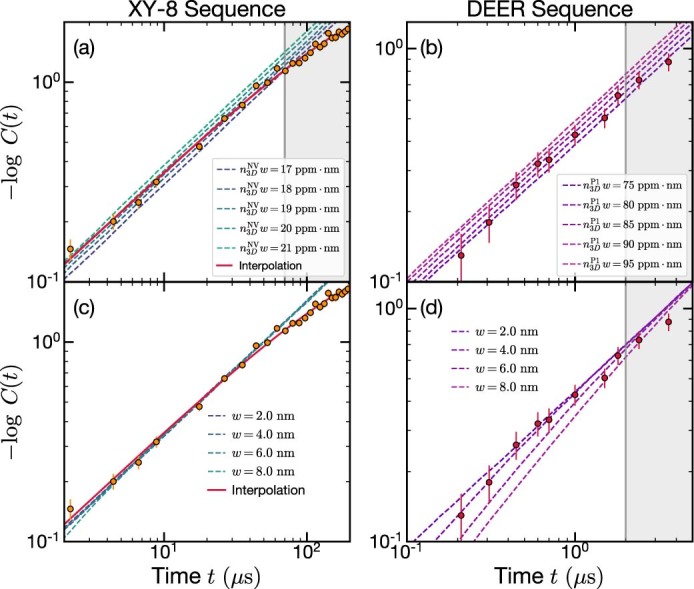
Defect densities. (a-b) NV and P1 areal densities. Computed dynamics (dashed lines) for early-time Ramsey decoherence caused by NV-NV interactions (a) and NV-P1 interactions (b), as a function of the NV areal density ${n}_{3{{{\rm{D}}}}}^{{{{\rm{NV}}}}} w$ and the P1 areal density ${n}_{3{{{\rm{D}}}}}^{{{{\rm{P}}}}1}w$, respectively. We compare these numerical results against the measured decoherence dynamics obtained via the XY-8 sequence (orange points, a) and the DEER sequence [after removing the NV contribution via the red interpolation in (a)] (purple points, b) to obtain the areal density of defects in sample S1. We estimate the areal density of NV centers to be ${n}_{3{{{\rm{D}}}}}^{{{{\rm{NV}}}}} w=19\pm 2\,{{{\rm{ppm}}}}\cdot {{{\rm{nm}}}}$ and the P1 density to be ${n}_{3{{{\rm{D}}}}}^{{{{\rm{P}}}}1} w=85\pm 10\,{{{\rm{ppm}}}}\cdot {{{\rm{nm}}}}$. At late times (grey shaded regions), the noise dynamics approach an incoherent random walk and should not be used to compute the density within this analysis, because the numerics do not include flip-flop interactions. (c-d) Effect of finite-thickness layer. At fixed areal density 19ppm ⋅ nm, choosing different layer widths *w* does not affect the computed dynamics (dashed curves, c). For higher density P1 centers ${n}_{3{{{\rm{D}}}}}^{{{{\rm{P}}}}1}=85\,{{{\rm{ppm}}}}\cdot {{{\rm{nm}}}}$, the finite thickness of the layer can induce a sizable effect on the DEER decoherence dynamics (purple points, d) at early times. Numerical calculations are plotted as dotted lines. Source data

We estimate the NV areal density in sample S1 via the XY-8 decoherence profile^62^, which is dominated by intragroup NV interactions (that is, within the NV group aligned with the applied magnetic field *B*) (Fig. 4a). We therefore treat the XY-8 data as a Ramsey measurement of the average NV–NV coupling, which we convert to a density using the dipolar interaction strength *J*_0_ = 2π × 52 MHz nm^3^. We compare the XY-8 data with numerically computed Ramsey decoherence, which we calculate as follows: We consider a central probe NV interacting with a bath of other NVs, placed randomly in a thin slab of thickness *w* with density ${n}_{{{{\rm{3D}}}}}^{{{{\rm{NV}}}}}/4$ (one NV group). After selecting a random spin configuration for the bath NVs, we compute the Ramsey signal $\sim \cos (\phi )$ for the probe NV. We then average over many such samples, and the resulting curve exhibits a stretched exponential decay of the form $C(t)=\mathrm{e}^{-{(t/{T}_{2})}^{2/3}}$. This functional form matches our expectation for the early-time ballistic regime (Table 1), because we have not included flip-flop dynamics in the numerical model. We treat the decoherence as arising only from intragroup Ising interactions, which is correct at short times when the NV centres are spin polarized. With the above prescription, we compute a set of Ramsey signals (Extended Data Fig. 1a, dashed lines) as a function of areal density ${n}_{{{{\rm{3D}}}}}^{{{{\rm{NV}}}}}w$, which we compare against the XY-8 data (Extended Data Fig. 1a, orange points). The estimated areal density is thus ${n}_{{{{\rm{3D}}}}}^{{{{\rm{NV}}}}} w =19\pm 2\,{{{\rm{ppm}}}}\, {{{\rm{nm}}}}$, corresponding to a density ${n}_{{{{\rm{3D}}}}}^{{{{\rm{NV}}}}}=3.2\pm 0.3\,{{{\rm{ppm}}}}$ assuming a layer with thickness of *w* = 6 nm.

*P1 density*

We estimate the P1 density by using a similar procedure but with DEER data instead of XY-8 data. We first remove the contribution due to NV–NV interactions from the DEER signal by subtracting an interpolation of the XY-8 data (Extended Data Fig. 1a) from the raw DEER data. Then, we compare the measured early-time dynamics with numerically computed curves for a range of P1 densities ${n}_{{{{\rm{3D}}}}}^{{{{\rm{P1}}}}}/3$ (Extended Data Fig. 1b). Here, we include a factor of 1/3 in the P1 density because the microwave tone *ω*_P1_ addresses only one-third of the P1 spins (the ‘P1-1/3 group’) in our DEER measurement, which are separated by ∼ 100 MHz from the four other groups due to the hyperfine interaction^63,64^. By comparing the data and theory curves, we estimate an areal density of ${n}_{{{{\rm{3D}}}}}^{{{{\rm{P1}}}}} w=85\pm 10\,{{{\rm{ppm}}}}\, {{{\rm{nm}}}} \approx 1.4(1)\times 1{0}^{-2}\,{{{{\rm{nm}}}}}^{-2}$.

At fixed areal density, the numerics indicate that the DEER decoherence profile depends on the layer thickness (Extended Data Fig. 1d). The same dependence is not present in the XY-8 dynamics due to the relatively small density of NV centres (Extended Data Fig. 1c). Although this method does not yield nanometre resolution, our observations are inconsistent with a layer with *w* > 6 nm, placing a more stringent bound on the thickness of the layer. The areal density ${n}_{{{{\rm{3D}}}}}^{{{{\rm{P1}}}}} w=85\pm 10\,{{{\rm{ppm}}}}\, {{{\rm{nm}}}}$ corresponds to ${n}_{{{{\rm{3D}}}}}^{{{{\rm{P1}}}}}=14\pm 2\,{{{\rm{ppm}}}}$, assuming a layer with thickness of *w* = 6 nm.

**Extended Data Fig. 2:**
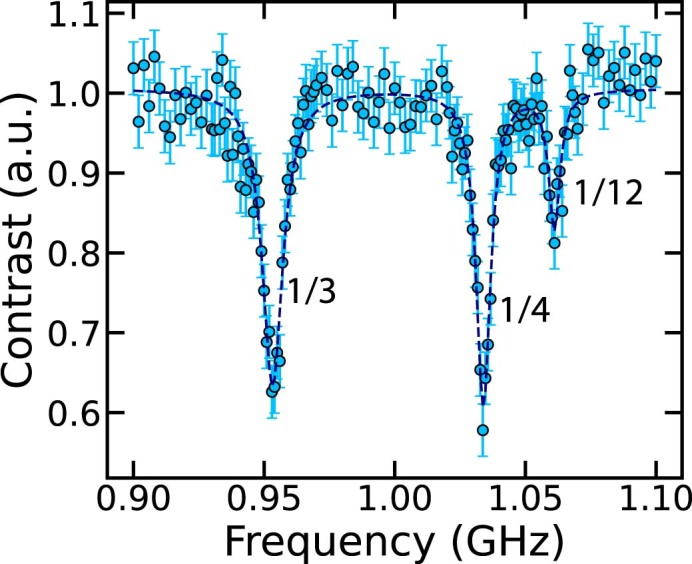
Relative density extraction for three of the five different P1 groups. The P1 spectrum is fit to a sum of three Lorentzian curves (dashed line). The relative areas of the three dips are 1 : 0.74 : 0.26, which is consistent with the expected ratio 1 : 0.75 : 0.25. Source data

Other spin 1/2 paramagnetic defects in diamond^65^ may have the same resonant frequency as the P1-1/3 group, causing a possible systematic error in our method for estimating the P1 density. To determine whether such defects are present in sample S1, we measured the P1 spectrum and compared the relative integrated areas under the peaks for the P1-1/3, 1/4 and 1/12 groups, thus obtaining an estimate of the relative densities between P1 groups. As shown in Extended Data Fig. 2, the results agree with the expected ratios 1:0.75:0.25 and are consistent with a negligible contribution of non-P1 defects to the DEER signal.

#### Sample S2

A detailed characterization of the three-dimensional sample S2 is given in ref. ^24^ (sample C041). Here, we describe the key properties relevant for the present study. The sample was grown by depositing a 32 nm diamond buffer layer, followed by a 500 nm nitrogen-doped layer (99%^15^N), and finished with a 50 nm undoped diamond capping layer. Vacancies were created by irradiating with 145 keV electrons at a dosage of 10^21^ cm^−2^, and vacancy diffusion was activated by annealing at 850 °C for 48 h in an Ar/Cl atmosphere. The resulting NV density is ∼0.4 ppm, obtained through instantaneous diffusion measurements^24^. The P1 density is measured to be ∼20 ppm through a modified DEER sequence^24^. The average spacing between P1 centres (∼4 nm) is much smaller than the thickness of the nitrogen-doped layer, ensuring three-dimensional behaviour of the spin ensemble.

#### Samples S3 and S4

Samples S3 and S4 used in this work are synthetic type Ib single/crystal diamonds (Element Six) with intrinsic substitutional ^14^N concentration of ∼100 ppm (calibrated with an NV linewidth measurement^63^). To create NV centres, the samples were first irradiated with electrons (2 MeV energy and 1 × 10^18^ cm^−2^ dosage) to generate vacancies. After irradiation, the diamonds were annealed in vacuum (∼10^−6^ Torr) with temperature >800 °C. The NV densities for both samples were measured to be ∼0.5 ppm using a spin-locking measurement^63^.

### Experimental methods

#### Experimental details for sample S1

The delta-doped sample S1 was mounted in a scanning confocal microscope. For optical pumping and readout of the NV centres, about 100 μW of 532 nm light was directed through an oil-immersion objective (Nikon Plan Fluor 100×, NA 1.49). The NV fluorescence was separated from the green 532 nm light by using a dichroic filter and collected on a fibre-coupled single-photon counter. A magnetic field *B* was produced by using a combination of three orthogonal electromagnetic coils and a permanent magnet, and aligned along one of the diamond crystal axes. The microwaves used to drive magnetic dipole transitions for both NV and P1 centres were delivered via an Omega-shaped stripline with typical Rabi frequencies of ∼2π × 10 MHz.

*DROID and Hartmann–Hahn sequences*

**Extended Data Fig. 3:**
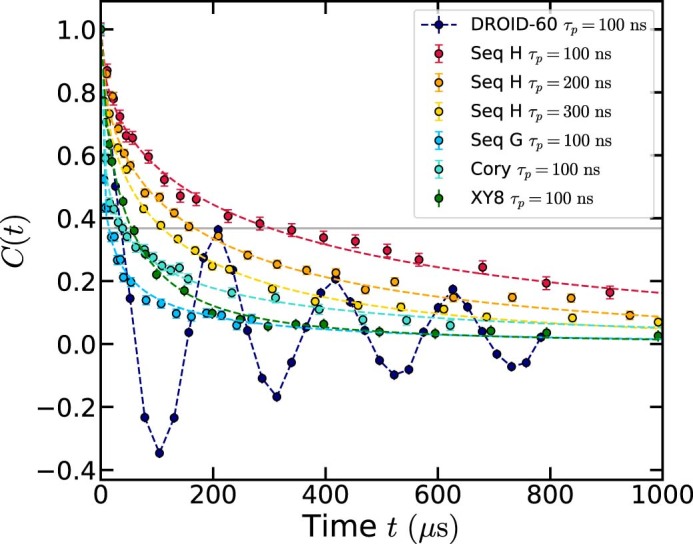
The measured decoherence profiles of variations on DROID-60. Due to imperfections in our composite microwave pulses, pulse error accumulates coherently in the DROID-60 (ref. ^62^) sequence and a pronounced oscillation is observed (purple points). To avoid such oscillations, we implement sequences that do not require composite pulses, see for example Seqs. A, H, G in Fig. 9 of ref. ^62^. The data exhibiting the longest coherence time (Seq. H, *τ*_*p*_ = 100ns, red points) are also shown in Fig. 4(a) of the main text. Source data

Here, we describe the pulse sequences used to perform the measurements shown in Fig. 4. In Fig. 4a, we compare the coherence times across different dynamical decoupling sequences, demonstrating that the longest coherence times are achieved when we decouple both on-site disorder and dipolar NV–NV dynamics using a DROID sequence proposed by Choi et al.^62^. To achieve the best decoupling, we experimented with a few variations on the well-known DROID-60 sequence^51^. These measurements are plotted in Extended Data Fig. 3. The DROID-60 data exhibit a pronounced coherent oscillation (purple points), which we attribute mainly to errors in composite pulses formed by sequential π/2 rotations along different axes. The data exhibiting the longest coherence time (red points) are obtained using so-called sequence H (fig. 9 of ref. ^62^). We hypothesize that sequence H behaves more predictably precisely because it eliminates composite pulses.

In Fig. 4b,c, we demonstrate polarization transfer between NV and P1 spins using a Hartmann–Hahn sequence. Following an initial π/2 pulse, the NV centres are spin-locked with Rabi frequency *Ω*_NV_, while the P1 centres are simultaneously driven with Rabi frequency *Ω*_P1_, for a duration *t*_s_. A final π/2 pulse is applied before detection. When the two Rabi frequencies are equal, that is, *Ω*_NV_ = *Ω*_P1_, the spins are resonant in the rotating frame, and spin-exchange interactions enhance the depolarization rate. The resonant depolarization data (Fig. 4b, green curve) are well fitted by the functional form

6 $$C(t)=\mathrm{e}^{-t/{T}_{1\rho }}\mathrm{e}^{-{(t/\tau )}^{D/2\alpha }},$$

where the first factor captures phonon-limited exponential decay and the second factor captures the independent depolarization channel driven by spin-exchange interactions, with stretch power *β* = 1/3 (ref. ^47^). We determine *T*_1*ρ*_ = 1.05(3) ms from the NV spin-locking measurement (Fig. 4b, orange curve).

#### Experimental details for sample S2

Sample S2 was mounted in a confocal microscope. For optical initialization and readout, about 350 μW of 532 nm light was directed through an air objective (Olympus UPLSA 40×, NA 0.95). The NV fluorescence was similarly separated from the 532 nm light by using a dichroic mirror and directed onto a fibre-coupled avalanche photodiode. A permanent magnet produced a field of about 320 G at the location of the sample. The field was aligned along one of the NV axes, and alignment was demonstrated by maximizing the ^15^N nuclear polarization^66^. Microwaves were delivered with a free-space rf antenna positioned over the sample.

#### Experimental details for samples S3 and S4

Samples S3 and S4 were mounted in a confocal microscope. For optical initialization and readout, about 3 mW of 532 nm light was directed through an air objective (Olympus LUCPLFLN, NA 0.6). The NV fluorescence was separated from the 532 nm light by using a dichroic mirror and directed onto a fibre-coupled photodiode (Thorlabs). The magnetic field was produced with an electromagnet with field strength of ∼174 G (∼275 G) for sample S3 (S4). The field was aligned along one of the NV axes, and microwaves were delivered using an Omega-shaped stripline with typical Rabi frequencies of ∼2π × 10 MHz.

#### Polychromatic drive

The polychromatic drive was generated by phase modulating the resonant P1 microwave tone^67^. A random array of phase jumps Δ*θ* was pre-generated and loaded onto an arbitrary waveform generator controlling the IQ modulation ports of a signal generator. The linewidth of the drive δ*ω* was controlled by fixing the s.d. of the phase jumps $\sigma =\sqrt{\updelta \omega \updelta t}$ in the pre-generated array, where 1/δ*t* = 1 gigasample per second was the sampling rate of the arbitrary waveform generator. The power in the drive was calibrated by measuring Rabi oscillations of the P1 centres without modulating the phase, that is, by setting δ*ω* = 0.

### Spin echo for samples S1 and S2 without polychromatic driving

**Extended Data Fig. 4:**
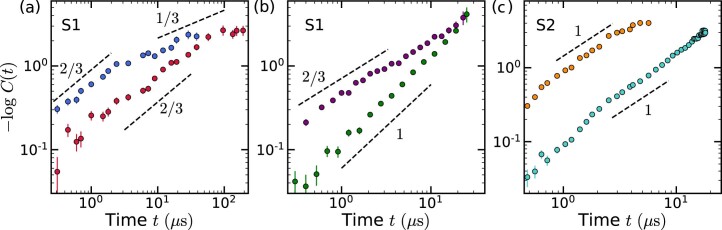
Undriven DEER and spin echo data. (a) In sample S1, with a clean surface, both the DEER (blue) and spin echo (red) data exhibit a stretch power *β* = 2/3. (b) After worsening the surface quality, the spin bath becomes noisier and we observe the expected *β* = 1 stretch power in the echo data (green); in the DEER data (purple) the correlation time *τ*_*c*_ increases but the stretch power is unchanged. (c) As in panel (a), the spin echo data (teal) for sample S2, presumably limited by NV-NV interactions rather than the bath, exhibit the same stretch power *β* = 1 as the DEER data. The DEER data in (a, c) are also plotted in Fig. 2(a) of the main text. Source data

In Section Characterizing microscopic spin-flip dynamics, we discussed spin echo measurements limited by NV–P1 interactions (as one would naively expect) and which exhibit an early-time stretch power of *β* = 3*D*/2*α* = 3/2. These measurements were performed on samples S3 and S4 that exhibit a P1-to-NV density ratio of ∼200. By contrast, spin echo measurements on samples S1 and S2, with P1-to-NV density ratios of ∼ 10 and ∼ 40, respectively, exhibit an early-time stretch of *β* = *D*/*α* (Extended Data Fig. 4), consistent with the prediction for a Ramsey measurement (Table 1). Here, we are discussing a ‘canonical’ spin echo measurement with no polychromatic drive (Fig. 2b, inset), and thus these data are not in contradiction with those presented in Fig. 3.

A possible explanation for the observed early-time stretch *β* = *D*/*α* is that the spin echo signal is limited by NV–NV interactions rather than by NV–P1 interactions. To understand this limitation, it is important to realize that the measured spin echo signal actually contains at least two contributions: (1) the expected spin echo signal from NV–P1 interactions, arising because the intermediate π pulse decouples the NVs from any quasi-static P1 contribution and (2) a Ramsey signal from NV interactions with other NVs, arising because these NVs are flipped together by the π pulse, and the intragroup Ising interactions are not decoupled.

Our hypothesis that NV–NV interactions limit the spin echo coherence in sample S1 is supported by the fact that a stretch power of *β* = 3*D*/2*α* = 1 can in fact be observed in spin echo data if the environment is made noisier, for example, by reversibly worsening the quality of the diamond surface (Extended Data Fig. 4b, green points). A subsequent three-acid clean restores the original *β* = 2/3 stretch power (Extended Data Fig. 4a, red points).

### Data analysis and fitting

#### Normalization of decoherence data

The coherence of the NV spins is read out via the population imbalance between $\{\left\vert 0\right\rangle ,\left\vert -1\right\rangle \}$ states. The maximum measured contrast ≲8% is proportional (not equal) to the normalized coherence *C*(*t*). To see a physically meaningful stretch power in our log–log plots of the data (Figs. 2 and 3), it is necessary to normalize the data by an appropriate value that captures our best approximation of the *t* = 0 time point for the DEER and spin echo measurements.

*Samples S1, S3 and S4: t* = *0 measurement*

**Extended Data Fig. 5:**
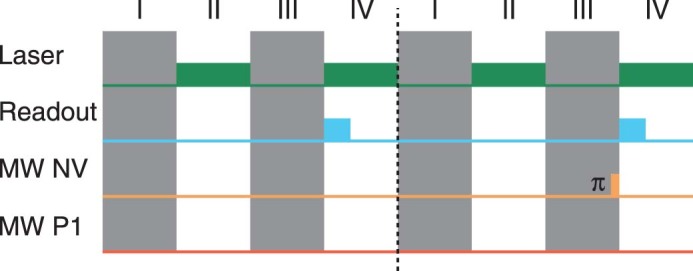
Experiment sequence schematic for differential measurement. The pulses for the laser (green), photodetector readout (blue), and microwaves addressing the NV (orange) and P1 (red) transitions are shown. The differential measurement subtracts the fluorescence obtained from two sequences (before and after the dotted vertical line), which are identical except for an additional π-pulse on the NV spins.

For a given pulse sequence (for example, Ramsey or spin echo) and fixed measurement duration *t*, we perform a differential readout of the populations in the $\left\vert 0\right\rangle$ and $\left\vert -1\right\rangle$ spin states of the NV, which mitigates the effect of NV and P1 charge dynamics induced by the laser initialization and readout pulses. As depicted schematically in Extended Data Fig. 5, we allow the NV charge dynamics to reach steady state (I) before applying an optical pumping pulse (II). Subsequently, we apply microwave pulses to both the NV and P1 spins (for example Ramsey or spin echo pulse sequences as shown in Figs. 2 and 3) (III). Finally, we detect the NV fluorescence (IV) to measure the NV population in $\left\vert 0\right\rangle$, obtaining a signal *S*_0_. We repeat the same sequence a second time, with one additional π pulse before detection to measure the NV population in $\left\vert -1\right\rangle$, obtaining a signal *S*_−1_. The raw contrast, *C*_raw_, at time *t* is then computed as *C*_raw_(*t*) ≡ [*S*_0_(*t*) − *S*_−1_(*t*)]/*S*_0_(*t*), and is typically ≲8%. We normalize the raw contrast to the *t* = 0 measurement to obtain the normalized coherence, *C*(*t*), defined above as

7 $$C(t)={C}_{{{{\rm{raw}}}}}(t)/{C}_{{{{\rm{raw}}}}}(t=0).$$

*Sample S2: t* = *0* *measurement*

For sample S2, we have an early-time, rather than a *t* = 0, measurement at *t* = 320 ns for spin echo and DEER sequences. Because the DEER signal decays on a much faster timescale than the spin echo signal, we normalize both datasets to the earliest-time spin echo measurement.

#### Data analysis for Fig. 3

We separate our discussion of the data analysis relevant to Fig. 3 into two parts. First, we discuss how comparing the *D* = 2 and *D* = 3 best fits to the DEER measurements enables us to identify the dimensionality of the underlying spin system. Second, armed with the fitted dimensionality, we fit spin echo and DEER measurements simultaneously to equation (5) to extract the correlation time *τ*_c_ of the P1 system. We note that, except for the *t* = 0 normalization point (Section Normalization of decoherence data in the Methods), we only consider data at times *t* > 0.5 μs, to mitigate any effects of early-time coherent oscillations caused by the hyperfine coupling between the NV and its host nitrogen nuclear spin.

*Determining the dimensionality of the system*

To determine the dimensionality of the different samples S1 and S2, we focus on the DEER signal, where the stretch power is given by *β* = *D*/*α* in the early-time ballistic regime and *β* = *D*/2*α* in the late-time random-walk regime. Employing both Gaussian and Markovian assumptions, a closed form for the decoherence can be obtained as

8 $${C}^{{{{\rm{DEER}}}}}(t)=\mathrm{e}^{-A{[{\chi }^{{{{\rm{DEER}}}}}(t)]}^{D/2\alpha }},$$

where *χ*^DEER^ is defined in equation (5) (Supplementary Information).

**Extended Data Fig. 6:**
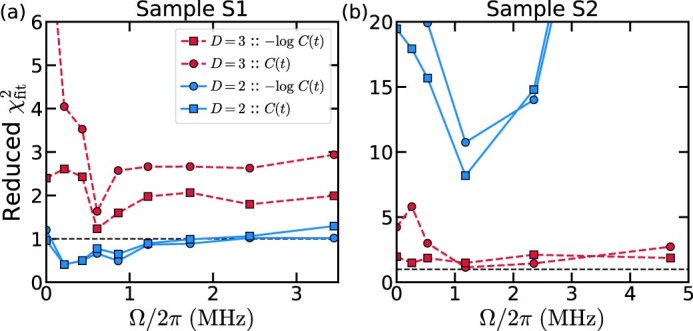
Reduced ${\chi }_{{{{\rm{fit}}}}}^{2}$ for fits to the DEER measurements on samples S1 (a) and S2 (b) for four different fit models. Reduced ${\chi }_{{{{\rm{fit}}}}}^{2}$ for fits to the DEER measurements on samples S1 (a) and S2 (b) for four different fit models. Source data

Armed with equation (8), we consider the decoherence dynamics for different powers of the polychromatic drive for both *D* = 2 and *D* = 3 (with *α* = 3, as per the dipolar interaction). We compare the reduced ${\chi }_{{{{\rm{fit}}}}}^{2}$ goodness-of-fit parameters for the two values of *D*, and demonstrate that the stretch power analysis above indeed agrees with the dimensionality that best explains the observed DEER data. Changing the dimension *D* does not change the number of degrees of freedom in the fit, so a direct comparison of ${\chi }_{{{{\rm{fit}}}}}^{2}$ is meaningful. Our results are summarized in Extended Data Fig. 6, where we observe that for sample S1 indeed the *D* = 2 fitting leads to a smaller ${\chi }_{{{{\rm{fit}}}}}^{2}$, while for sample S2 the data are best captured by *D* = 3 (Extended Data Fig. 6). Independently fitting both the extracted signal *C*(*t*) as well to its negative logarithm $-\log C(t)$ yields the same conclusions. This analysis complements the discussion above in terms of the early-time and late-time stretch power of the decay.

*Extracting the correlation time τ*_c_

Having determined the dimensionality of samples S1 and S2, we now turn to characterizing the correlation times of the P1 spin systems in these samples. To robustly extract *τ*_*c*_, we perform a simultaneous fit to both the DEER signal with equation (8) and the spin echo signal with

9 $${C}^{{{{\rm{SE}}}}}(t)=\mathrm{e}^{-A{[{\chi }^{{{{\rm{SE}}}}}(t)]}^{D/2\alpha }},$$

assuming a single amplitude *A* and correlation time *τ*_c_ for both normalized signals. Here, *χ*^DEER/SE^ depends on *τ*_c_ as defined in equation (5).

To carefully evaluate the uncertainty in the extracted correlation time, we take particular care to propagate the uncertainty in the *t* = 0 data used to normalize the raw contrast, that is, *C*_raw_(*t* = 0) (Section Normalization of decoherence data in the Methods). Owing to the two normalization methods for samples S1 and S2 (Section Normalization of decoherence data in the Methods), we estimate the uncertainty in two different ways:

- For samples S1, S3 and S4, we consider fluctuations of the normalization value, *C*_raw_(*t* = 0), by ±10%. This is meant to account for a possible effect of the hyperfine interaction in this data point, as well as any additional systematic error.
- For sample S2, we first compute a linear interpolation of the early-time spin echo decoherence to *t* = 0. We then sample the normalization uniformly between this extrapolated value and the earliest spin echo value.

By sampling over the possible values of *C*_raw_(*t* = 0), we build a distribution over the extracted values of *τ*_*c*_ fitting to both the coherence, *C*(*t*), and its logarithm, $-\log C(t)$. The reported values in Fig. 3d,e correspond to the mean and s.d. evaluated over this distribution.

**Extended Data Fig. 7:**
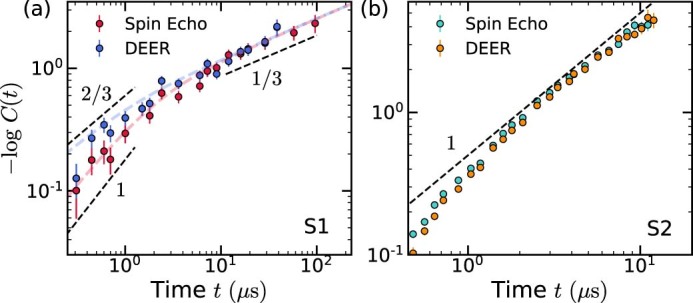
DEER and spin echo for sample S1 (a) and sample S2 (b), under a fast incoherent drive. **DEER and spin echo for sample S1 (a) and sample S2 (b), under a fast incoherent drive**. The Rabi frequencies for panels (a) and (b) are Ω = 2*π* × 3.45MHz and Ω = 2*π* × 4.05MHz, respectively. The data obtained for sample S2 plotted in (b) does not exhibit the correct “random-walk” regime stretch power of 1/2, even though the DEER and spin echo signals overlap at all measured times. Source data

We end this section by commenting that, as the drive strength is reduced, the spin echo signal looks increasingly similar to the undriven spin echo data (Extended Data Fig. 4), that is, the early-time stretch changes from *β* = 3*D*/2*α* to *β* = *D*/*α*. Our explanation for this observed stretch is given in Section Spin echo for samples S1 and S2 without polychromatic driving in the Methods. The deviation from the expected functional form for the decoherence leads to a large uncertainty in the extracted correlation time. The data also deviate from the model for larger drive strengths, for example, *Ω* = 2π × 4.05 MHz, δ*ω* = 2π × 20 MHz, where our assumption that δ*ω* ≫ Ω is no longer valid (Extended Data Fig. 7).

## Online content

Any methods, additional references, Nature Portfolio reporting summaries, source data, extended data, supplementary information, acknowledgements, peer review information; details of author contributions and competing interests; and statements of data and code availability are available at 10.1038/s41567-023-01944-5.

## Supplementary information

Supplementary Information Supplementary discussion.

## Data Availability

Data supporting the findings of this paper are available from the corresponding authors upon request. Source data are provided with this paper. Source data for Figs. 1–4 and Extended Data Figs. 1–7 are provided with this paper.

## Source data

Source Data Fig. 1 Source data.
Source Data Fig. 2 Source data.
Source Data Fig. 3 Source data.
Source Data Fig. 4 Source data.
Source Data Extended Data Fig. 1 Source data.
Source Data Extended Data Fig. 2 Source data.
Source Data Extended Data Fig. 3 Source data.
Source Data Extended Data Fig. 4 Source data.
Source Data Extended Data Fig. 6 Source data.
Source Data Extended Data Fig. 7 Source data.

## Code availability

Code developed for the data analysis and visualization is available from the corresponding author upon request.

## References

[1] Purcell, E. M. in *Confined Electrons and Photons* (eds Burstein, E. & Weisbuch, C.) 839–839 (Springer, 1995).

[2] Viola L, Knill E, Lloyd S (1999). Dynamical decoupling of open quantum systems. Phys. Rev. Lett..

[3] Houck A (2008). Controlling the spontaneous emission of a superconducting transmon qubit. Phys. Rev. Lett..

[4] De Lange G, Wang Z, Riste D, Dobrovitski V, Hanson R (2010). Universal dynamical decoupling of a single solid-state spin from a spin bath. Science.

[5] Tyryshkin AM (2012). Electron spin coherence exceeding seconds in high-purity silicon. Nat. Mater..

[6] Klauder J, Anderson P (1962). Spectral diffusion decay in spin resonance experiments. Phys. Rev..

[7] Schweiger, A. & Jeschke, G. *Principles of Pulse Electron Paramagnetic Resonance* (Oxford University Press on Demand, 2001).

[8] Kofman A, Kurizki G (2000). Acceleration of quantum decay processes by frequent observations. Nature.

[9] Romach Y (2015). Spectroscopy of surface-induced noise using shallow spins in diamond. Phys. Rev. Lett..

[10] Kleppner D (1981). Inhibited spontaneous emission. Phys. Rev. Lett..

[11] Kotler S, Akerman N, Glickman Y, Keselman A, Ozeri R (2011). Single-ion quantum lock-in amplifier. Nature.

[12] Bar-Gill N (2012). Suppression of spin-bath dynamics for improved coherence of multi-spin-qubit systems. Nat. Commun..

[13] Herzog B, Hahn EL (1956). Transient nuclear induction and double nuclear resonance in solids. Phys. Rev..

[14] Kubo, R., Toda, M. & Hashitsume, N. *Statistical Physics II: Nonequilibrium Statistical Mechanics*, Vol. 31 (Springer Science & Business Media, 2012).

[15] Salikhov K, Dzuba S-A, Raitsimring AM (1981). The theory of electron spin-echo signal decay resulting from dipole–dipole interactions between paramagnetic centers in solids. J. Magn. Reson. (1969).

[16] Chiba M, Hirai A (1972). Electron spin echo decay behaviours of phosphorus doped silicon. J. Phys. Soc. Jpn..

[17] Altman E, Demler E, Lukin MD (2004). Probing many-body states of ultracold atoms via noise correlations. Phys. Rev. A.

[18] Hofferberth S (2008). Probing quantum and thermal noise in an interacting many-body system. Nat. Phys..

[19] Fel’dman EB, Lacelle S (1996). Configurational averaging of dipolar interactions in magnetically diluted spin networks. J. Chem. Phys..

[20] Choi S (2017). Observation of discrete time-crystalline order in a disordered dipolar many-body system. Nature.

[21] Sushkov A (2014). Magnetic resonance detection of individual proton spins using quantum reporters. Phys. Rev. Lett..

[22] Ohno K (2012). Engineering shallow spins in diamond with nitrogen delta-doping. Appl. Phys. Lett..

[23] McLellan CA (2016). Patterned formation of highly coherent nitrogen-vacancy centers using a focused electron irradiation technique. Nano Lett..

[24] Eichhorn TR, McLellan CA, Bleszynski Jayich AC (2019). Optimizing the formation of depth-confined nitrogen vacancy center spin ensembles in diamond for quantum sensing. Phys. Rev. Mater..

[25] Yao NY, Zaletel MP, Stamper-Kurn DM, Vishwanath A (2018). A quantum dipolar spin liquid. Nat. Phys..

[26] Chomaz L (2019). Long-lived and transient supersolid behaviors in dipolar quantum gases. Phys. Rev. X.

[27] Semeghini G (2021). Probing topological spin liquids on a programmable quantum simulator. Science.

[28] Anderson PW, Weiss PR (1953). Exchange narrowing in paramagnetic resonance. Rev. Mod. Phys..

[29] Georgescu, I. M., Ashhab, S. & Nori, F. Quantum simulation. *Rev. Mod. Phys.* **86**, 153 (2014).

[30] Engelsberg M, Lowe I, Carolan J (1973). Nuclear-magnetic-resonance line shape of a linear chain of spins. Physical Review B.

[31] Cho G, Yesinowski JP (1996). H and 19f multiple-quantum NMR dynamics in quasi-one-dimensional spin clusters in apatites. J. Phys. Chem..

[32] Cho H, Ladd TD, Baugh J, Cory DG, Ramanathan C (2005). Multispin dynamics of the solid-state NMR free induction decay. Phys. Rev. B.

[33] Mims W (1968). Phase memory in electron spin echoes, lattice relaxation effects in CaWO_4_: Er, Ce, Mn. Phys. Rev..

[34] Abe E, Itoh KM, Isoya J, Yamasaki S (2004). Electron-spin phase relaxation of phosphorus donors in nuclear-spin-enriched silicon. Phys. Rev. B.

[35] Zhong M (2015). Optically addressable nuclear spins in a solid with a six-hour coherence time. Nature.

[36] de Sousa R, Sarma SD (2003). Theory of nuclear-induced spectral diffusion: spin decoherence of phosphorus donors in si and gaas quantum dots. Phys. Rev. B.

[37] Wang Z-H, Takahashi S (2013). Spin decoherence and electron spin bath noise of a nitrogen-vacancy center in diamond. Phys. Rev. B.

[38] Bauch E (2020). Decoherence of ensembles of nitrogen-vacancy centers in diamond. Phys. Rev. B.

[39] Hanson R, Dobrovitski V, Feiguin A, Gywat O, Awschalom D (2008). Coherent dynamics of a single spin interacting with an adjustable spin bath. Science.

[40] Ernst RR (1966). Nuclear magnetic double resonance with an incoherent radio-frequency field. J. Chem. Phys..

[41] Hu P, Hartmann SR (1974). Theory of spectral diffusion decay using an uncorrelated-sudden-jump model. Phys. Rev. B.

[42] Cucchietti FM, Paz JP, Zurek WH (2005). Decoherence from spin environments. Phys. Rev. A.

[43] de Sousa, R. in *Electron Spin Resonance and Related Phenomena in Low-Dimensional Structures* (ed Fanciulli, M.) 183–220 (Springer, 2009).

[44] Yang W, Ma W-L, Liu R-B (2017). Quantum many-body theory for electron spin decoherence in nanoscale nuclear spin baths. Rep. Prog. Phys..

[45] Kogan, S. *Electronic Noise and Fluctuations in Solids* (Cambridge Univ. Press, 2008).

[46] Witzel W, Sarma SD (2006). Quantum theory for electron spin decoherence induced by nuclear spin dynamics in semiconductor quantum computer architectures: spectral diffusion of localized electron spins in the nuclear solid-state environment. Phys. Rev. B.

[47] Choi J (2017). Depolarization dynamics in a strongly interacting solid-state spin ensemble. Phys. Rev. Lett..

[48] Zhidomirov G, Salikhov K (1969). Contribution to the theory of spectral diffusion in magnetically diluted solids. Sov. J. Exp. Theor. Phys..

[49] Glasbeek M, Hond R (1981). Phase relaxation of photoexcited triplet spins in cao. Phys. Rev. B.

[50] Witzel WM, Carroll MS, Cywiński Ł, Sarma SD (2012). Quantum decoherence of the central spin in a sparse system of dipolar coupled spins. Phys. Rev. B.

[51] Zhou H (2020). Quantum metrology with strongly interacting spin systems. Phys. Rev. X.

[52] Takahashi S, Hanson R, Van Tol J, Sherwin MS, Awschalom DD (2008). Quenching spin decoherence in diamond through spin bath polarization. Phys. Rev. Lett..

[53] Belthangady C (2013). Dressed-state resonant coupling between bright and dark spins in diamond. Phys. Rev. Lett..

[54] Laraoui A, Meriles CA (2013). Approach to dark spin cooling in a diamond nanocrystal. ACS Nano.

[55] Bruin A. Many-body delocalization in a strongly disordered system with long-range interactions: finite-size scaling. *Phys. Rev. B* **91**, 094202 (2015).

[56] Yao NY (2014). Many-body localization with dipoles. Phys. Rev. Lett..

[57] Hartmann S, Hahn E (1962). Nuclear double resonance in the rotating frame. Phys. Rev..

[58] Cappellaro P, Ramanathan C, Cory DG (2007). Dynamics and control of a quasi-one-dimensional spin system. Phys. Rev. A.

[59] Lukin DM, Guidry MA, Vučković J (2020). Integrated quantum photonics with silicon carbide: challenges and prospects. PRX Quantum.

[60] Rosskopf T (2014). Investigation of surface magnetic noise by shallow spins in diamond. Phys. Rev. Lett..

[61] Syntek. Products 1: various industrial diamonds. *Syntek* http://www.syntek.co.jp/en/products/ (2023).

[62] Choi J (2020). Robust dynamic Hamiltonian engineering of many-body spin systems. Phys. Rev. X.

[63] Zu C (2021). Emergent hydrodynamics in a strongly interacting dipolar spin ensemble. Nature.

[64] Hall LT (2016). Detection of nanoscale electron spin resonance spectra demonstrated using nitrogen-vacancy centre probes in diamond. Nat. Commun..

[65] Grinolds M (2014). Subnanometre resolution in three-dimensional magnetic resonance imaging of individual dark spins. Nat. Nanotechnol..

[66] Jacques V (2009). Dynamic polarization of single nuclear spins by optical pumping of nitrogen-vacancy color centers in diamond at room temperature. Phys. Rev. Lett..

[67] Joos, M., Bluvstein, D., Lyu, Y., Weld, D. M. & Jayich, A. B. Protecting qubit coherence by spectrally engineered driving of the spin environment. *npj Quantum Inf.* **8**, 47 (2022).

