# Supplementary material for: Probing many-body dynamics in a two-dimensional dipolar spin ensemble
Source: Nat Phys. 2023 Mar 16;19(6):836–44. doi: 10.1038/s41567-023-01944-5 (PMC10264245; doi:10.1038/s41567-023-01944-5)
Supplement: Supplementary file 1 — Supplementary discussion. [file 41567_2023_1944_MOESM1_ESM.pdf]

nature physics

Article https://doi.org/10.1038/s41567-023-01944-5

Probing many-body dynamics in a two-dimensional dipolar spin ensemble

In the format provided by the authors and unedited

Supplementary Information:
Probing many-body dynamics in a two dimensional dipolar spin ensemble

In this supplement, we provide additional details on the results presented in the "Theoretical framework for decoherence dynamics induced by many-body noise" section of the main text. In Sec. I, we derive the decoherence dynamics within a semi-classical description that assumes Gaussian fluctuations. This presentation collects many ideas found in previous works [1–14]. We aim to provide a clear, self-contained discussion and to derive a highly general expression for the decoherence dynamics which can be applied to a range of spin systems. In Sec. II, we provide a complementary quantum mechanical description of the same decoherence dynamics, which enables us to frame the distinction between Gauss-Markov and telegraph noise in terms of the delocalization of quantum information.

Throughout this supplement, we consider the same setup as in the main text; namely, a probe spin-1/2 $\hat{s}_p$ interacting via long-range, $1/r^\alpha$, Ising interactions with system spins $\hat{s}_i$:

$$H_z = \sum_i \frac{J_z g_i}{r_i^\alpha} \hat{s}_p^z \hat{s}_i^z, \tag{S1}$$

where we have explicitly separated the overall interaction strength J_z from any angular dependence $g_i \sim \mathcal{O}(1)$.

We are interested in the decoherence dynamics where the probe spin is initially prepared along the $\hat{x}$-axis of the Bloch sphere and the Ising interactions [Eqn. S1] cause its precession in the equatorial plane. The coherence of the spin is defined as the (normalized) projection of the spin into the $\hat{x}$-axis at a later time, $C(t) = 2\langle \hat{s}_p^x(t) \rangle$.

During the evolution, a decoupling sequence can be applied which flips the probe spin. In the rotating frame, the decoupling sequence can be thought of as changing the sign of the Ising interaction. This can be straightforwardly accounted for by introducing a function, $\eta(t';t)$, which captures the sign of H_z at time t' for a pulse sequence of duration t. For example, for a Ramsey measurement $\eta_{\mathrm{R}}(t';t) = 1$, while for spin echo

$$\eta_{\mathrm{SE}}(t';t) = \begin{cases} 1 & 0 < t' < t/2 \\ -1 & t/2 < t' < t. \end{cases} \tag{S2}$$

We note that this analysis focuses only on Ising interactions and thus ignores the effects of depolarization on the decoherence dynamics. This is a natural assumption for NV-P1 systems where the two spin defects are far detuned ($\sim$ GHz) and inter-species spin-exchange interactions ($\sim$ MHz) are highly off-resonant and suppressed [15].

I. DERIVATION OF THE DECOHERENCE DYNAMICS ASSUMING GAUSS-MARKOV NOISE

In this section we analyze the decoherence problem by treating the system spins as fluctuating classical variables $\hat{s}_i^z(t) \to s_i^z(t)$. As such, instead of analysing the details of the dynamics of $\hat{s}_i^z$, we can relate the decoherence of the probe spin to the statistical properties of the fluctuating classical variables $s_i^z(t)$.

Within this framework, every trajectory of $s_i^z(t')$ induces a precession angle $\phi(t)$. The observed coherence is then given by the ensemble average of the coherence over all trajectories:

$$C = 2\langle \hat{s}_p^x(t) \rangle = \left\langle \mathrm{Re}\left[e^{-i\phi(t)} \right] \right\rangle, \quad \text{with} \quad \phi(t) = \sum_j \frac{J_z g_j}{r_j^\alpha} \int_0^t \eta(t';t) s_i^z(t') dt', \tag{S3}$$

where $\langle \ldots \rangle$ denotes the average over the ensemble of trajectories.

In order to proceed from Eqn. S3 into a closed form solution for the decoherence dynamics, we must relate the system dynamics to the statistical properties of $s_i^z(t)$ and $\phi(t)$; for simplicity, and following previous literature [1, 2, 10, 16–18], we assume that both $\phi(t)$ and $s_i^z(t)$ are accurately captured by Gauss-Markov processes. We postpone a detailed discussion about the validity of this assumption to a later section. This simplification allows us to obtain the

Sequence	$\eta(t';t)$	$\chi(t)$	Short-time $t \ll \tau_c$	Long-time $t \gg \tau_c$
Ramsey (DEER)	1	$2\tau_c t - 2\tau_c^2\left(1 - \mathrm{e}^{-\frac{t}{\tau_c}}\right)$	$t^2 - \frac{t^3}{3\tau_c}$	$2\tau_c t - 2\tau_c^2$
Spin Echo	$\begin{cases} 1 & 0 \le t' < t/2 \\ -1 & t/2 \le t' < t \end{cases}$	$2\tau_c t - 2\tau_c^2\left(3 + \mathrm{e}^{-\frac{t}{\tau_c}} - 4\mathrm{e}^{-\frac{t}{2\tau_c}}\right)$	$\frac{t^3}{6\tau_c}$	$2\tau_c t - 6\tau_c^2$
XY-8	$\begin{cases} 1 & (m-\frac{1}{4})\tau_p \le t' < (m+\frac{1}{4})\tau_p \\ -1 & (m+\frac{1}{4})\tau_p \le t' < (m+\frac{3}{4})\tau_p \end{cases}$	$\frac{\tau_p^2}{12\tau_c}t$	$\frac{\tau_p^2}{12\tau_c}t$	$\frac{\tau_p^2}{12\tau_c}t$

TABLE S1. Expressions of $\eta(t';t)$ and $\chi(t)$ for Ramsey/DEER, spin echo, and XY-8. In XY-8, we assume the inter-pulse spacing $\tau_p \ll \tau_c$.

decoherence dynamics in terms of the variance of $\phi(t)$:

$$C(t) = \langle \mathrm{Re}[e^{-i\phi(t)}]\rangle = e^{-\langle\phi^2\rangle/2} = \exp\left\{-\frac{1}{2}\left\langle\left|\sum_i \frac{J_z g_i \int_0^t \eta(t';t) s_i^z(t')dt'}{r_i^\alpha}\right|^2\right\rangle\right\}$$
$$= \prod_i \exp\left\{-\frac{1}{2}\left[\frac{J_z|g_i|\chi(t)^{\frac{1}{2}}}{2r_i^\alpha}\right]^2\right\}. \tag{S4}$$

Here, we assume that each spin is independent and define $\chi(t)$ as follows:

$$\chi(t) \equiv 4\left\langle\left[\int_0^t \eta(t';t)s_i^z(t')dt'\right]^2\right\rangle = \int_0^t dt' \int_0^t dt'' \eta(t';t)\eta(t'';t)\left\langle 4s_i^z(t')s_i^z(t'')\right\rangle. \tag{S5}$$

The assumption of Gauss-Markov noise enables us to write down the two-point correlation function in terms of a decaying exponential with time scale τ_c:

$$\xi(\tau) \equiv \langle 4s_i^z(t)s_i^z(t+\tau)\rangle = \mathrm{e}^{-|\tau|/\tau_c}. \tag{S6}$$

The factor of 4 ensures that the correlation function $\xi(\tau)$ is normalized for spin-1/2 particles when $\tau = 0$. Depending on the specific pulse sequence applied to the system spins, captured by $\eta(t';t)$, we can analytically obtain the expression for $\chi(t)$ [Table S1].

A few remarks are in order. First, $\chi(t)$ has an intuitive and straightforward interpretation in Fourier space. Defining $f(\omega;t)$ as the Fourier transform of $\eta(t';t)$ and $S(\omega)$ as the Fourier transform of $\xi(\tau)$, $\chi(t)$ can be rewritten as:

$$\chi(t) = \int d\omega\ |f(\omega;t)|^2 S(\omega), \tag{S7}$$

which recovers Eqn. 2 of the main text. In this language, the role of the pulse sequence becomes clear: given the noise spectrum $S(\omega)$ of the spin dynamics $s_i^z(t)$, the pulse sequence acts as a filter function $f(\omega;t)$. Changing the pulse sequence modifies the probe spin's sensitivity to different frequency components of the noise.

Second, for a single realization, the time-dependence of the decoherence dynamics is entirely determined by $\chi(t)$. If $\chi(t)$ only depends on τ_c and the pulse sequence, the decoherence dynamics is not sensitive to the dimensionality of the spin system. The ability to probe the dimensionality of the spin system arises from the interplay between the positional disorder and the power-law interactions, as discussed in detail in the next section.

I.1. Average over positional randomness

Until now, we have considered only the role of dynamical fluctuations in our analysis of the decoherence. In this subsection, we review the effect of positional disorder on the observed decoherence decay. Following Ref. [7], we compute the positional disorder average by first considering N system spins occupying a volume V in D dimensions,

and explicitly performing the volume integration of each spin as follows:

$$\begin{aligned} C(t) &= \int \cdots \int \frac{d^D\vec{r}_1}{V}\frac{d^D\vec{r}_2}{V}\cdots\frac{d^D\vec{r}_N}{V}\prod_{i=1}^{N}\exp\left\{-\frac{1}{2}\left[\frac{J_z|g_i|\chi(t)^{\frac{1}{2}}}{2r_i^\alpha}\right]^2\right\} \\ &= \left[\int \frac{d^D\vec{r}}{V}\exp\left\{-\frac{1}{2}\left[\frac{J_z|g|\chi(t)^{\frac{1}{2}}}{2r^\alpha}\right]^2\right\}\right]^N = \left[1-\frac{1}{N}\frac{N}{V}\int d^D\vec{r}\left(1-\exp\left\{-\frac{1}{2}\left[\frac{J_z|g|\chi(t)^{\frac{1}{2}}}{2r^\alpha}\right]^2\right\}\right)\right]^N. \end{aligned} \tag{S8}$$

This last equality gives us the limit definition of the exponential ($\lim_{N\to\infty}(1+x/N)^N = e^x$) and, in the thermodynamic limit ($N, V \to \infty$ with fixed density $n \equiv \frac{N}{V}$), is given by:

$$\begin{aligned} C(t) &= \exp\left\{-n\int d^D\vec{r}\left(1-\mathrm{e}^{-\frac{1}{2}\left[\frac{J_z|g|\chi(t)^{\frac{1}{2}}}{2r^\alpha}\right]^2}\right)\right\} = \exp\left\{-n\int_0^\infty dr\int d\Omega\left(1-\mathrm{e}^{-\frac{1}{2}z^2}\right)r^{D-1}\right\} \\ &= \exp\left\{-\frac{n}{\alpha}\left(\frac{J_z\chi(t)^{\frac{1}{2}}}{2}\right)^{\frac{D}{\alpha}}\left[\int_0^\infty dz\left(1-\mathrm{e}^{-\frac{1}{2}z^2}\right)z^{-\frac{D}{\alpha}-1}\right]\left[\int d\Omega\ |g|^{\frac{D}{\alpha}}\right]\right\} \\ &= \exp\left\{-\frac{nDA_D}{\alpha}\left[-\frac{\Gamma(-\frac{D}{2\alpha})}{2^{\frac{D}{2\alpha}+1}}\right]\left[\frac{\overline{g}J_z\chi(t)^{\frac{1}{2}}}{2}\right]^{\frac{D}{\alpha}}\right\}, \end{aligned} \tag{S9}$$

where we make the substitution $z = \frac{J_z|g|\chi(t)^{\frac{1}{2}}}{2r^\alpha}$, $A_D = \frac{\pi^{\frac{D}{2}}}{\Gamma(\frac{D}{2}+1)}$ is the volume of a D-dimensional unit ball, and $\overline{g} = \left(\frac{\int |g|^{\frac{D}{\alpha}}d\Omega}{\int d\Omega}\right)^{\frac{\alpha}{D}}$ is the averaged angular dependence over a D-dimensional solid angle.

We note that the integral converges only when $2\alpha > D$. This condition captures the physical intuition that, for very long-range interactions (small α), the probe spin interacts strongly with an extensive number of spins. More precisely, the number of spins at distance r from the probe increases as $r^{D-1}dr$, while their contribution scales as $r^{-2\alpha}$. The variance of the phase ϕ, at any fixed time, is then given by $\int_0^R dr\ r^{D-2\alpha-1}$, which precisely diverges with increasing R when $2\alpha > D$. In this regime, which does not apply for our measurements, the standard deviation of $\phi(t)$ becomes unbounded in the thermodynamic limit, the Gaussian approximation $C = e^{-\langle\phi^2\rangle/2}$ no longer applies, and a more careful analysis is required.

Combining the results in Table S1 and Eqn. S9, we obtain the analytical form of the decoherence signal as measured in our system. In both the short-time and the long-time limits, $\chi(t)$ has a simple power-law dependence on time t. In each limit, the form of the decay profile is a simple stretched exponential, from which we can also obtain the decay timescale as a function of the defect density. For example, for the early-time Ramsey decay, we have:

$$\begin{aligned} C^{\mathrm{Ramsey}}(t \ll \tau_c) &= \exp\left\{-\frac{nDA_D}{\alpha}\left[-\frac{\Gamma(-\frac{D}{2\alpha})}{2^{\frac{D}{2\alpha}+1}}\right]\left[\frac{\overline{g}J_z t}{2}\right]^{\frac{D}{\alpha}}\right\} = \exp\left\{-\left[\left(-\frac{\Gamma(-\frac{D}{2\alpha})}{2^{\frac{3D}{2\alpha}+1}}\frac{DA_D}{\alpha}\right)^{\frac{\alpha}{D}} n^{\frac{\alpha}{D}}\overline{g}J_z t\right]^{\frac{D}{\alpha}}\right\} \\ &= \exp\left\{-\left(\frac{t}{T_2^{\mathrm{Ramsey}}}\right)^{\frac{D}{\alpha}}\right\}, \end{aligned} \tag{S10}$$

where

$$T_2^{\mathrm{Ramsey}} = \left[\left(-\frac{\Gamma(-\frac{D}{2\alpha})}{2^{\frac{3D}{2\alpha}+1}}\frac{DA_D}{\alpha}\right)^{\frac{\alpha}{D}} n^{\frac{\alpha}{D}}\overline{g}J_z\right]^{-1} \propto n^{-\frac{\alpha}{D}}. \tag{S11}$$

Similarly, we can obtain the analytical forms for other pulse sequences and in different time regimes, with all results summarized in Table S2.

Here, let us emphasize the role of positional disorder in determining the shape of the decoherence decay profile, as highlighted by the differences between Eqns. S4 and S9. To explain this difference, it is important to emphasize that

	Early-time $t \ll \tau_c$		Late-time $t \gg \tau_c$	
	Stretch power	Decay timescale	Stretch power	Decay timescale
Ramsey (DEER)	$\frac{D}{\alpha}$	$\left(K\overline{g}n^{\frac{\alpha}{D}}J_z\right)^{-1}$	$\frac{D}{2\alpha}$	$\left(\frac{1}{2\tau_c}\right)\left(K\overline{g}n^{\frac{\alpha}{D}}J_z\right)^{-2}$
Spin Echo	$\frac{3D}{2\alpha}$	$(6\tau_c)^{\frac{1}{3}}\left(K\overline{g}n^{\frac{\alpha}{D}}J_z\right)^{-\frac{2}{3}}$	$\frac{D}{2\alpha}$	$\left(\frac{1}{2\tau_c}\right)\left(K\overline{g}n^{\frac{\alpha}{D}}J_z\right)^{-2}$
XY8	$\frac{D}{2\alpha}$	$\left(\frac{12\tau_c}{\tau_p^2}\right)\left(K\overline{g}n^{\frac{\alpha}{D}}J_z\right)^{-2}$	$\frac{D}{2\alpha}$	$\left(\frac{12\tau_c}{\tau_p^2}\right)\left(K\overline{g}n^{\frac{\alpha}{D}}J_z\right)^{-2}$

TABLE S2. Ensemble averaged decay profiles for Ramsey/DEER, spin echo, and XY-8 pulse sequences; $\overline{g}$ is the averaged angular dependence, and $K = \frac{1}{2}\left[-\frac{DA_D}{\alpha}\frac{\Gamma(-\frac{D}{2\alpha})}{2^{\frac{D}{2\alpha}+1}}\right]^{\frac{\alpha}{D}}$ is a dimensionless constant only depending on D and α.

the main contribution to the average coherence is dominated by different positional configurations at different times. For example, at early times, only the configurations with very nearby spins are able to decohere, while configurations with distant spins have yet to contribute to the signal. Similarly, at late times, the configurations with fast decay rates have fully decohered, so the signal is dominated by configurations where the spins are far away from the probe and decoherence is slow. This explains why the stretch power is determined by both dimensionality (which determines how many system spins can be close to the probe) and the exponent of the power-law interactions (which determines how fast those configurations decohere).

By contrast, if the system spins are positioned on a regular lattice, the decay profile no longer exhibits a distribution of time scales and thus follows the shape of the single positional realization case [Eqn. S4]. In particular, for a regular lattice, the spin echo always decays as a stretched exponential with a stretch exponent of 3 (Table S1), independent of both D and α.

I.2. Dependence of coherence time on dimension

Thus far, we have focused primarily on the functional form of the decoherence dynamics of the probe spin. Another important insight from Eqn. S9, also highlighted in Table S2, is that the dimensionality D also affects the scaling of the coherence time with density as $T_2 \sim n^{-\alpha/D}$, where $n \equiv N/V$.

Naively, for fixed α, this scaling may appear to imply that the coherence time in lower-dimensional samples decays faster than in higher-dimensional samples. However, to directly compare the coherence time across dimensions, one must find a setting where the different densities (which carry different units) can be related. In practice, our delta-doped layer provides such a setting; namely, because it has a finite thickness w, we can relate the two-dimensional areal density to the three-dimensional volume density $n_{\mathrm{2D}} = n_{\mathrm{3D}} \cdot w$. At low densities $n_{\mathrm{3D}}^{-1/3} \gg w$, the thin layer can be treated as a two-dimensional system. Within this setting, the coherence time of the probe spin in the two-dimensional layer will be longer, since it is surrounded by a much smaller number of system spins. Thin layers which do not satisfy this condition, i.e. which contain large spin densities $n_{\mathrm{3D}}^{-1/3} \leq w$, can no longer be considered two-dimensional and recover the three-dimensional scaling.

II. QUANTUM DESCRIPTION

In Sec. I, we assumed that the spin dynamics can be captured by classical Gaussian random variables. In general this need not be true, and one should treat the spin dynamics quantum-mechanically. The goal of this section is to perform such an analysis and use it to highlight how the distinction between Gauss-Markov and telegraph noise can be cast as a property of the scrambling dynamics of the system spin operators $\hat{s}_i^z(t)$.

The full Hamiltonian H governing the dynamics in our experiments is composed of the Ising interaction H_z between the probe spin and the systems spins, as well as H_s which determines the interactions between the system spins.

Including the effect of the pulse sequence yields

$$H_{\rm tot}(t') = \eta(t';t)H_z + H_{\rm s}. \tag{S12}$$

The initial density matrix of the system immediately encodes the ensemble of possible initial states. In particular, we consider initial state of the system to be a fully mixed state for the system's spins, while the probe spin is initialized in the $\hat{x}$ direction. This corresponds to the following density matrix:

$$\rho(t=0) = \frac{\mathbb{1}}{\mathcal{N}_s} \otimes \left(\frac{|\uparrow\rangle + |\downarrow\rangle}{\sqrt{2}}\right)\left(\frac{\langle\uparrow| + \langle\downarrow|}{\sqrt{2}}\right), \tag{S13}$$

where $\mathcal{N}_s$ is the size of the Hilbert space of the spin system.

The probe coherence then becomes:

$$C(t) = 2\mathrm{Tr}\left[\hat{s}_p^x U(t)\rho U^\dagger(t)\right], \quad \text{where} \quad U(t) = \mathcal{T}\exp\left[-i\int_0^t dt' H_{\rm tot}(t')\right]. \tag{S14}$$

Because the dynamics conserve $\hat{s}_p^z$, the unitary operator U can be divided into two operators $U_{\uparrow/\downarrow}$, which act only on the system spins and compute their evolution conditioned on the state of the probe:

$$U(t) = U_\uparrow(t) \otimes |\uparrow\rangle\langle\uparrow| + U_\downarrow(t) \otimes |\downarrow\rangle\langle\downarrow|, \tag{S15}$$

where

$$U_\uparrow(t) = \mathcal{T}\mathrm{e}^{-i\int_0^t [H_{\rm s}+\eta(t';t)\sum_i \frac{J_z g_i}{2r_i^\alpha}\hat{s}_i^z]dt'}, \qquad U_\downarrow(t) = \mathcal{T}\mathrm{e}^{-i\int_0^t [H_{\rm s}-\eta(t';t)\sum_i \frac{J_z g_i}{2r_i^\alpha}\hat{s}_i^z]dt'}. \tag{S16}$$

The coherence can then be written in terms of $U_{\uparrow/\downarrow}(t)$ acting on the spin system:

$$C(t) = \frac{1}{\mathcal{N}_s}\mathrm{Tr}\left[U_\uparrow(t)U_\downarrow^\dagger(t) + U_\downarrow(t)U_\uparrow^\dagger\right] = \frac{2}{\mathcal{N}_s}\mathrm{Re}\left(\mathrm{Tr}\left[U_\uparrow(t)U_\downarrow^\dagger(t)\right]\right) \tag{S17}$$

The dynamics $U_{\downarrow/\uparrow}(t)$ remain very complex because they include a contribution from both the system's dynamics and the probe-system coupling. To understand how the probe spin is sensitive to the spin dynamics generated by the many-body interactions H_s, we move to the interaction frame of H_s by making the following substitution:

$$U_{\uparrow/\downarrow}(t) = U_0(t)\tilde{U}_{\uparrow,\downarrow}(t), \quad \text{with} \quad U_0(t) = \mathrm{e}^{-itH_{\rm s}}, \tag{S18}$$

where

$$\tilde{U}_{\uparrow/\downarrow} = \mathcal{T}\mathrm{e}^{\mp i\int_0^t \eta(t';t)\sum_i \frac{J_z g_i}{2r_i^\alpha}\hat{\tilde{s}}_i^z(t')dt'}, \quad \text{and} \quad \hat{\tilde{s}}_i^z(t) = U_0^\dagger(t)\hat{s}_i^z(t)U_0(t). \tag{S19}$$

In this frame, the two evolution operators lose an explicit reference to H_s at the expense of the spin operators $\hat{s}_i^z$ becoming time-dependent. The resulting coherence remains in the same form, but with the unitaries now referring to the interaction frame:

$$C(t) = \frac{2}{\mathcal{N}_s}\mathrm{Re}\left(\mathrm{Tr}\left[\tilde{U}_\uparrow(t)\tilde{U}_\downarrow^\dagger(t)\right]\right) \tag{S20}$$

Note that Eqn. S20 is formally similar to Eqn. S3. Especially, replacing the quantum operator $\hat{\tilde{s}}_i^z(t)$ with a classical variable immediately reduce this equation to Eqn. S3.

While Eqn. S20 already averages over the possible initial states of $\hat{s}_i^z$, one must independently average the signal over the different spin ensembles (which arise, for example, from different positions of the spins, coupling to a polychromatic driving field or to other classical fluctuating degrees of freedom, etc.) While these two kinds of averages (one from different configurations of the many-body system and the other from the randomness of $H_{\rm s}$) can lead to the same auto-correlator $\xi(t) \propto \langle\hat{\tilde{s}}_i^z(t)\hat{\tilde{s}}_i^z(0)\rangle$, they are essential for determining higher order moments of the distribution and, thus, whether a telegraph or a continuous (Gaussian) random variable is a good description of the many-body noise of $\hat{s}_i^z$ and thus the decoherence signal of the probe spin.

II.1. Understanding decoherence dynamics in different physical scenarios

While Eqn. S20 provides the generic expression for the decoherence dynamics of a spin coupled to a dynamical system, it is intractable to directly compute the decoherence profile from this equation except in specific cases. Nevertheless, such solvable points can provide important intuition for the conditions under which Eqn. S20 reduces to a semi-classical description with either Gauss-Markov or telegraph noise. In the following subsections, we provide two instructive examples where an explicit computation can be performed, and the relationship between the nature of the system and its noise properties is made clear.

II.1.1. Probe coupled to a single spin evolving under an external drive

First, we consider the case where the noise that decoheres the probe is generated by a single system spin, whose dynamics are controlled by an *external random* drive. In this case, the interaction Hamiltonian is given by:

$$H_{\mathrm{s}} = \Omega[\hat{s}_i^x \cos\theta(t) + \hat{s}_i^y \sin\theta(t)] \tag{S21}$$

where Ω characterizes the strength of the drive and $\theta(t)$ is a time-dependent phase. The presence of such a time-dependent phase mirrors to the polychromatic drive described in the main text—$\theta(t)$ is chosen to follow a Gaussian stochastic process (see Methods, Sec. IV.4) [19], and is randomized across different runs of the experiment. Crucially, for each run of the experiment, the dynamics induced by H_{s} generate a particular trajectory around the Bloch sphere without any loss of single-particle coherence. As a result, the continuous spin rotation leads to a continuous change in the strength of the noise generated—this leads to the natural description of $s_i^z(t)$ as a continuous classical variable.

We emphasize that, within this framework, there is a single phase accumulated due to the noise for the particular driving $\theta(t)$. As a result, to obtain Gaussian-distributed noise, one must additionally average over different instances of $\theta(t)$. In the experiment this corresponds exactly to the polychromatic drive, where the phase accumulation rate in each experimental run is random and changes continuously in time.

II.1.2. Probe coupled to a system strongly interacting with a large bath

We now turn to the opposite limit, where the noise is generated by spins coupled to a Markovian bath. The dynamics of $\hat{s}_i^z(t)$ can be thought to undergo spontaneous emission and absorption of photons/phonons—starting in either the state $|\uparrow\rangle$ or $|\downarrow\rangle$, the system undergoes quantum jumps into the opposite state at a rate given by $1/\tau_c$ [20]. In this intuitive picture, the decoherence of the probe spin should be evaluated by averaging over all the possible quantum jump trajectories of the system spins—this precisely corresponds to a telegraph-like classical noise.

We can make this picture more precise, using Eqn. S19 as well as intuition from the perspective of operator dynamics. In particular, if the operator $\hat{\tilde{s}}_i^z(t)$ spreads across a large number of degrees of freedom much faster than the interaction time scale with the probe spin, then the probe spin is interacting with independent, commuting operators at different times, $[\hat{\tilde{s}}_z^i(t), \hat{\tilde{s}}_z^i(t')] = 0$. This immediately leads to two consequences: (i) the time-ordering operator in Eqn. S19 acts trivially on the exponential, and the eigenvalues of the exponential are simply given by the exponential of the eigenvalues of $\int_0^t \eta(t')\hat{\tilde{s}}_i^z(t')dt'$; (ii) $\hat{\tilde{s}}_i^z(t)$ can be simultaneously diagonalized for different times.

The latter fact enables a very simple analysis of the dynamics. To see this more easily, let us simplify our problem by dividing the time evolution into M independent blocks of duration $\delta t = t/M$:

$$\tilde{U}_{\uparrow/\downarrow} = \prod_j \exp\left[\mp i\eta(j\delta t;t)\sum_i \frac{J_z g_i}{2r_i^\alpha}\hat{\tilde{s}}_i^z(j\delta t)\right], \tag{S22}$$

where $\hat{\tilde{s}}_i^z(j\delta t)$ commute with one another for different i and time $j\delta t$. Because there is a single eigenbasis $|\mu\rangle$ that diagonalizes all these operators, when computing $\langle\mu|\,\tilde{U}_{\uparrow/\downarrow}\,|\mu\rangle$, the operators can be substituted by the corresponding eigenvalues $\lambda_\mu^i(j\delta t)$, and the exponent simply becomes the sum of individual contributions. Crucially, since $\hat{s}_z^2(t) = 1/4$, $\lambda_\mu^i(t)$ can only be $\pm 1/2$ [21]. The last necessary ingredient is the understanding of how $\lambda_\mu^i(j\delta t)$ at different times relate with one another. In general, this depends on the details of the dynamics. But, owing to the size of the Hilbert space, $2\mathcal{N}_s$, one can take a statistical approach to this question. Namely, over the ensemble of eigenstates and operators, changing the sign of the eigenstate is expected to follow a Poisson process with time scale τ_c, $\langle\lambda_\mu^i(t)\lambda_\mu^i(t')\rangle \propto \mathrm{e}^{-|t-t'|/\tau_c}$. We note that this correlation decay can be exactly recast as an exponential decay correlation function for the spin

operators. The dynamics of each $\lambda^i_\mu(t)$ can then be understood as either a single quantum jump trajectory (in the quantum language), or a single classical telegraph noise realization (in the classical description). Summing over all the eigenstates $|\mu\rangle$ completes the trace operation in Eqn. S20 and yields the final coherence of the probe spin.

Here, we hasten to emphasize that unlike the single driven spin case, one does not have to compute an average over different trajectories of the spin dynamics, that is already incorporated within the trace operation and the sum over the different eigenstates. This contrasts with the single spin example, where an explicit averaging over the driving fields was necessary to obtain the distribution of accumulated phases.

II.1.3. Spin coupled to a generic many-body interacting system

Taking the above two examples into consideration, whether a generic many-body system is described by the Gauss-Markov or the Telegraph random variable is determined by the speed of the operator spreading. If the spreading of the operator is slow, the dynamics of $\hat{s}^i_z(t)$ remain constrained to a few sites throughout the measurement duration and the system appears coherent (leading to continuous Gaussian noise). If the spreading of the operator is fast, $\hat{s}^i_z(t)$ quickly spreads across many spins and the rest of the system acts as an effective Markovian bath, leading to telegraph noise.

In our disordered, strongly-interacting system, we conjecture that disorder leads to the slow spread of $\hat{s}^z_i$, and the decay of the auto-correlator $\langle \hat{s}^z_i(0)\hat{s}^z_i(t)\rangle$ mostly results from the different trajectories of local dynamics (originating from different H_s owing to different initial configurations of the bath spins). This is consistent with our experimental observation of the spin-echo decay stretch power $\beta = 3D/2\alpha$ for a three-dimensional dipolar ensemble, and is characteristic of the Gaussian noise model.

[1] V. V. Dobrovitski, A. E. Feiguin, R. Hanson, and D. D. Awschalom, Physical Review Letters **102**, 237601 (2009).
[2] W. Yang, W.-L. Ma, and R.-B. Liu, Reports on Progress in Physics **80**, 016001 (2017), arXiv:1607.03993.
[3] P. W. Anderson and P. R. Weiss, Rev. Mod. Phys. **25**, 269 (1953).
[4] J. Klauder and P. Anderson, Physical Review **125**, 912 (1962).
[5] P. Hu and S. R. Hartmann, Phys. Rev. B **9**, 1 (1974).
[6] K. Salikhov, S.-A. Dzuba, and A. M. Raitsimring, Journal of Magnetic Resonance (1969) **42**, 255 (1981).
[7] E. B. Fel'dman and S. Lacelle, The Journal of chemical physics **104**, 2000 (1996).
[8] F. M. Cucchietti, J. P. Paz, and W. H. Zurek, Phys. Rev. A **72**, 052113 (2005).
[9] R. de Sousa, Electron Spin Resonance and Related Phenomena in Low-Dimensional Structures , 183–220 (2009).
[10] Z.-H. Wang, G. De Lange, D. Ristè, R. Hanson, and V. Dobrovitski, Physical Review B **85**, 155204 (2012).
[11] R. Kubo, M. Toda, and N. Hashitsume, *Statistical physics II: nonequilibrium statistical mechanics*, Vol. 31 (Springer Science & Business Media, 2012).
[12] L. Cywiński, R. M. Lutchyn, C. P. Nave, and S. Das Sarma, Phys. Rev. B **77**, 174509 (2008).
[13] Z.-H. Wang and S. Takahashi, Physical Review B **87**, 115122 (2013).
[14] M. Glasbeek and R. Hond, Physical Review B **23**, 4220 (1981).
[15] C. Zu, F. Machado, B. Ye, S. Choi, B. Kobrin, T. Mittiga, S. Hsieh, P. Bhattacharyya, M. Markham, D. Twitchen, A. Jarmola, D. Budker, C. R. Laumann, J. E. Moore, and N. Y. Yao, "Emergent hydrodynamics in a strongly interacting dipolar spin ensemble," (2021), arXiv:2104.07678 [quant-ph].
[16] B. Herzog and E. L. Hahn, Phys. Rev. **103**, 148 (1956).
[17] R. Hanson, V. Dobrovitski, A. Feiguin, O. Gywat, and D. Awschalom, Science **320**, 352 (2008).
[18] G. De Lange, Z. Wang, D. Riste, V. Dobrovitski, and R. Hanson, Science **330**, 60 (2010).
[19] M. Joos, D. Bluvstein, Y. Lyu, D. M. Weld, and A. B. Jayich, "Protecting qubit coherence by spectrally engineered driving of the spin environment," (2021), arXiv:2101.09654 [quant-ph].
[20] P. Meystre and M. Sargent, *Elements of Quantum Optics*, 4th ed. (Springer-Verlag, Berlin Heidelberg, 2007).
[21] As long as the eigenspectrum of the original local operator is discrete, so will $\lambda_\mu(t)$; as a result, we expect the same telegraph noise description in the context of higher spin systems.
